# Deep spectral–spatial learning for robust bruise detection and internal damage estimation in apples using visible/near-infrared hyperspectral imaging

**DOI:** 10.3389/fpls.2026.1967857

**Published:** 2026-10-27

**Authors:** Jiwon Ryu, Chang-Hyup Lee, Sang-Yeon Kim, Sungjay Kim, Minhyun Kim, Dong Heun Sung, Suk-Ju Hong, Ghiseok Kim

**Affiliations:** 1 Department of Biosystems Engineering, Seoul National University, Seoul, Republic of Korea; 2 Research Institute of Agriculture and Life Sciences, Seoul National University, Seoul, Republic of Korea; 3 Integrated Major in Global Smart Farm, Seoul National University, Seoul, Republic of Korea; 4 Department of Smart Bio-Industrial Mechanical Engineering, Kyungpook National University, Daegu, Republic of Korea

**Keywords:** apple, bruise detection, hyperspectral imaging, nondestructive evaluation, postharvest quality, three-dimensional convolutional neural network (3D CNN)

## Abstract

Bruises are common postharvest defects of apples whose appearance varies with surface location, impact severity, and time after impact, complicating visual detection particularly during the early stages of bruising. To address this challenge, four three-dimensional (3D) U-Net variants of spectral–spatial encoders were compared for bruise detection from visible/near-infrared (Vis/NIR) hyperspectral images. Furthermore, features learned by the bruise detector were reused to classify impact level and estimate internal cross-sectional bruise area. Controlled bruises were induced at three impact levels and multiple surface locations on 87 Fuji apples. Hyperspectral images were acquired from six views over 18 days of storage, resulting in 870 image cubes with 1,676 annotated bruise instances. Bruise depth and internal cross-sectional area of browned tissue were measured by destructive sectioning. Among the four models, the Hybrid 3D–2D U-Net achieved the highest test bounding box *F*1 score of 0.98 and a mask Dice score of 0.86. The model outperformed baseline detectors using inputs with three channels derived from principal component and band-ratio transformations, particularly for early, mild, and peripheral bruises, maintaining the *F*1 score above 0.98. Half-precision inference preserved both accuracy metrics at 87.63 frames per second. For the bruise severity estimation, models incorporating pretrained encoder features of the bruise detection model achieved a test macro-averaged *F*1 score of 0.83 for impact level classification and a test Pearson *r* of 0.82 for internal cross-sectional bruise area estimation, outperforming the baseline models based on the mean reflectance spectrum. These results indicated that deep spectral–spatial learning supported robust bruise detection while extending hyperspectral assessment from bruise localization to estimation of internal damage severity.

## 1 Introduction

Bruises are among the most frequent forms of mechanical damage observed during the postharvest handling of apples (*Malus domestica*) and are a major cause of quality loss along the supply chain (Opara and Pathare, 2014; Lin et al., 2023). Impact received during harvesting, transportation, and sorting damages the subcutaneous flesh, and the damaged tissue browns and softens through the oxidation of phenolic compounds by polyphenol oxidase (Diels et al., 2017). Damaged tissues are also more vulnerable to microbial infection, and the region affected by enzymatic browning has been reported to expand during storage (Çelik et al., 2021; Xu et al., 2024). Bruises formed within the first day after the impact often show internal browning without a visible change on the peel, so they are difficult to identify by visual inspection or conventional red, green, and blue (RGB) images (ElMasry et al., 2008; Li et al., 2025). Rapid, nondestructive detection of bruises together with assessment of their severity is therefore of considerable importance for maintaining the quality of apples.

Hyperspectral imaging has been widely adopted for nondestructive fruit quality evaluation because it acquires spatial and spectral information simultaneously across a range of wavelengths, capturing changes that are difficult to observe with conventional color imaging (Kim et al., 2001; Lorente et al., 2012; Manley, 2014). Hyperspectral imaging has therefore been an established approach for bruise detection in apples, and its usefulness has long been attributed to the near-infrared response of the damaged tissue. Lu (2003) identified the 1,000–1,340 nm range as effective for apple bruise detection using principal component and minimum-noise-fraction transforms, and Mehl et al. (2004) applied an asymmetric second-difference method based on the chlorophyll absorption band at 685 nm. Saeys et al. (2008) reported that the absorption and scattering properties of apple tissue beyond 700 nm were suited to probing subsurface structural change, and wavelengths in the red-edge and near-infrared regions have accordingly been identified as effective for detecting bruises on apples (Xing et al., 2005; Wang and ElMasry, 2010).

Previous studies on bruise detection in apples using hyperspectral imaging were commonly performed with hyperspectral cubes reduced to a small number of two-dimensional (2D) representations, including principal component analysis (PCA) images and band-ratio images, followed by segmentation using thresholds to locate bruised regions (Che et al., 2018; Du et al., 2020; Tian et al., 2023). Che et al. (2018) trained a random forest classifier using pixel spectra from manually defined bruised and sound regions and applied it to the unlabeled region between them to extract the complete bruise area, whereas Tian et al. (2023) combined a two-band ratio image with improved watershed segmentation and implemented the method in a multispectral imaging system. However, variance in the reflectance signal of the bruised regions caused by the curvature of the fruits was reported to interfere with bruise detection near the fruit periphery (Xing et al., 2005; Li et al., 2025; Wang et al., 2026). Surface features such as the stem and calyx were also reported to resemble defects and complicate inspection of the whole fruit (Fan et al., 2022). Moreover, spectral responses of the bruised tissues were reported to vary with the severity of the impact and the elapsed time after the impact (Wang and ElMasry, 2010; Zhang et al., 2021), which could limit the consistency of the segmentation of bruised pixels using a fixed threshold across these conditions.

To improve robustness to variations in bruise appearance and imaging artifacts, more recent studies adopted deep learning approaches based on the selected band images or principal component images derived from the entire hyperspectral cube. Pang et al. (2022) used PCA within three spectral regions to select characteristic wavelengths and applied YOLOv3 to the corresponding band images, correctly detecting all 110 bruises in the test set with an *F*1 score of 1.00 at 68 frames per second while avoiding false detections caused by specular highlights. Zhang et al. (2024) selected three short-wave infrared bands using the successive projections algorithm and PCA, enhanced bruise contrast through pseudo-color and band-ratio transformations, and localized early bruises using a lightweight FP-YOLOv5 model with a recognition rate of 95% at 130 frames per second. Other recent approaches incorporated spectral information more directly from the original hyperspectral cube, through three-dimensional (3D) spectral–spatial learning or learning across multiple selected bands. Both 2D and 3D CNNs were compared for image-level classification of bruised oranges (Pourdarbani et al., 2023), and a 3D spectral–spatial CNN classified plum cubes reduced by PCA as sound or bruised and by elapsed time after impact (Shanthini et al., 2026). In the case of apples, a 3D convolutional backbone was combined with a region-based detection head to localize early bruises (Liu et al., 2025), while a CNN–Transformer model classified mean spectra from predefined regions of interest using 18 selected bands (Liu et al., 2026). However, these image-level classification and region-based detection approaches did not distinguish bruises from sound tissue at a pixel level, limiting further analysis for estimating the damage severity of the bruised region.

Studies on hyperspectral images primarily focused on detecting the bruised region itself (Wieme et al., 2022; Grabska et al., 2023), while the effects of impact magnitude and elapsed time on the spectral response were generally evaluated in separate analyses based mainly on mean reflectance spectra (Wang and ElMasry, 2010; Zhang et al., 2021). Recent models based on spectral imaging combined bruise segmentation or sample classification with prediction of elapsed time or experimentally defined bruise level (Fang et al., 2025; Shanthini et al., 2026), and an apple model trained using three impact levels performed binary classification of mean spectra from predefined regions of interest (Liu et al., 2026). These prediction targets were categorical experimental conditions and did not use continuous measurements of internal tissue damage as reference values. Internal bruise extent has been quantified by destructive sectioning or X-ray computed tomography (Diels et al., 2017; Sidhu et al., 2023; Wood et al., 2024), but these methods are unsuitable for rapid inline inspection. Consequently, it remained unclear whether spatially resolved spectral features learned for bruise localization could classify impact level and estimate measured internal damage while bruise location and elapsed time varied together.

A pixel-wise prediction of the bruise masks from spectrally resolved hyperspectral cubes requires the extracted spectral–spatial features to be converted into spatial segmentation maps. U-Net has been widely used as a multiscale 2D encoder–decoder network with skip connections for pixel-wise segmentation of RGB images, and 3D U-Net extended its convolutional operations to 3D inputs for volumetric segmentation (Ronneberger et al., 2015; Çiçek et al., 2016). For hyperspectral pixel classification, HybridSN used 3D convolutions to learn joint spectral–spatial features from local hyperspectral patches and subsequent 2D convolutions to learn higher-level spatial features while reducing model complexity relative to an all-3D CNN (Roy et al., 2020). HybridSN performed patch-based classification of hyperspectral pixels, whereas hybrid 3D–2D U-Net architectures used 3D encoding and feature projection followed by 2D decoding to generate spatial segmentation maps from volumetric inputs (Ushinsky et al., 2021). These approaches provided a basis for combining 3D spectral–spatial feature extraction with 2D mask reconstruction for efficient pixel-wise bruise detection from hyperspectral images.

Accordingly, this study aimed to develop a deep spectral–spatial framework for robust bruise segmentation and further estimation of the internal damage of the bruises from visible/near-infrared (Vis/NIR) hyperspectral images. Specifically, three objectives were addressed:

1. Four 3D U-Net variants with spectral–spatial encoders were trained and compared for direct bruise mask prediction from hyperspectral reflectance cubes, including a Hybrid 3D–2D U-Net and two compact 3D U-Net variants.
2. Detection performance was compared with YOLO11s baselines across multiple radial locations, impact levels, and storage periods of the bruise instances.
3. Encoder features of the 3D U-Net model were reused and evaluated for impact level classification and internal cross-sectional bruise area estimation against baselines based on mean spectra.

## 2 Materials and methods

### 2.1 Experimental design and sample preparation

The experimental procedure comprised three stages (**Figure 1**), which were (1) preparation of apple samples and controlled bruise induction at different positions along the stem–calyx direction, (2) sequential hyperspectral image acquisition while each apple was rotated about the stem–calyx axis during cold storage, and (3) destructive measurement of internal browning at each time point. The rotation allowed the same bruise to appear at different radial positions on the visible apple surface.

**Figure 1:**
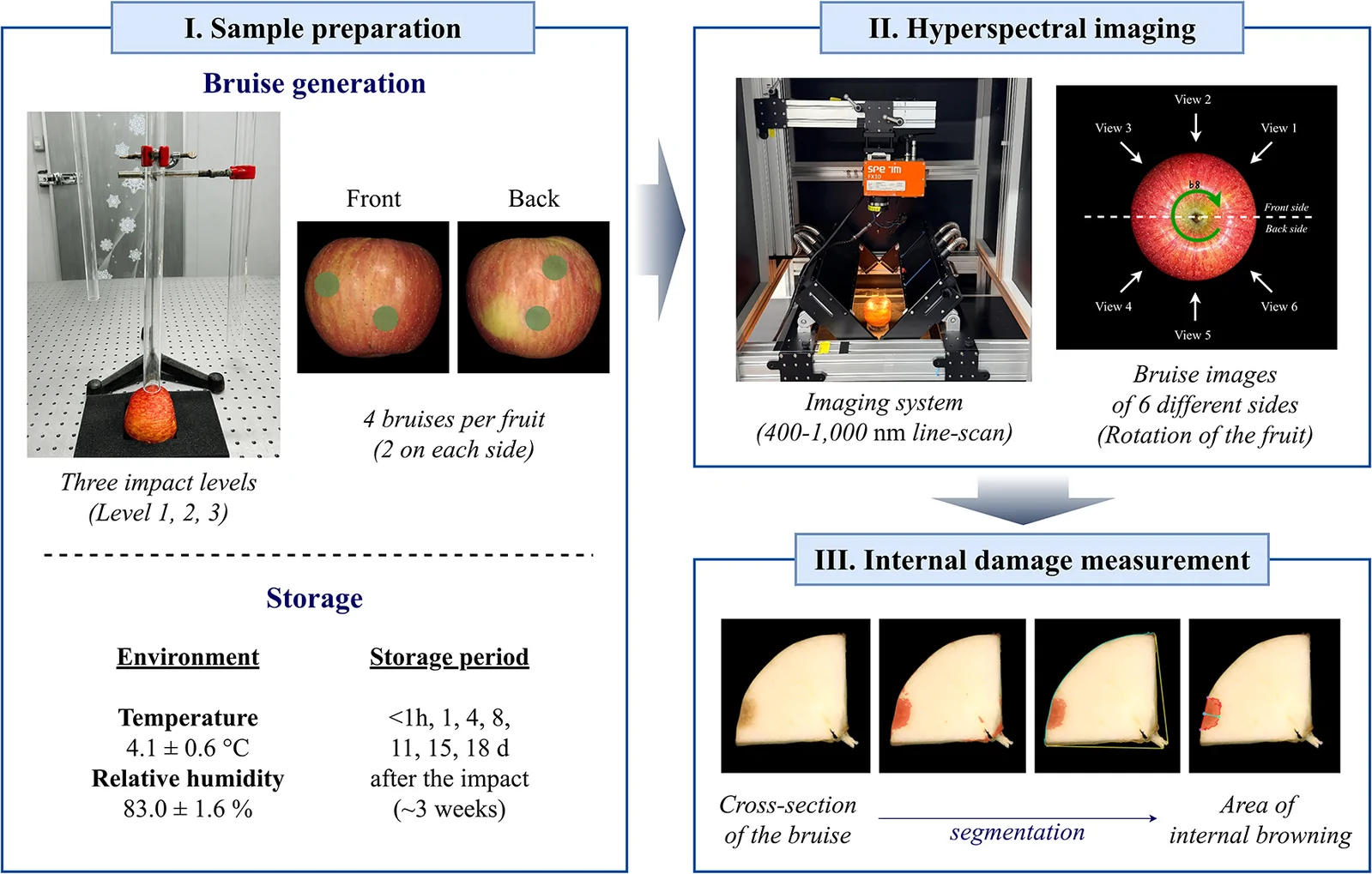
Overview of the experimental procedure used to induce and evaluate bruises on apples. Bruises were induced at three impact levels and at different positions along the stem–calyx direction. The apples were stored under refrigerated conditions and sampled at predefined time points. Hyperspectral images were acquired while each apple was sequentially rotated about the stem–calyx axis manually, allowing the same bruise to be recorded at different radial positions on the apple surface. Imaging was followed by destructive measurement of internal browning.

A total of 87 Fuji apples (*Malus domestica*) harvested in 2025 were obtained from an agricultural products processing center of Chungju-si, Chungcheongbuk-do, Republic of Korea. Fruits free from visible surface defects or pre-existing bruises were selected, and the mean weight of the apples was 257 g ± 7 g with a range of 242–273 g. Controlled bruises were induced by dropping a steel ball vertically through a transparent guide tube positioned above the apple surface, mostly two bruises on each side of the fruit, yielding four bruises per apple. Three impact levels were applied, and the impact energy was calculated as the kinetic energy of the ball immediately before contacting the fruit according to Equation 1 (Nturambirwe et al., 2020):

(1) E=mgh

where *E* is the impact energy, *m* is the ball mass, *g* is the gravitational acceleration (9.81 m/s^2^), and *h* is the drop height. The impact energies were 0.147 J for Level 1 (30 g ball dropped from 50 cm), 0.294 J for Level 2 (30 g ball dropped from 100 cm), and 0.539 J for Level 3 (110 g ball dropped from 50 cm), which were assigned to 29, 30, and 28 apples, respectively. After the bruise induction, the apples were stored in a cold storage chamber maintained at 4.1 °C ± 0.6 °C and 83.0% ± 1.6% relative humidity, logged at 30 min intervals using a temperature and humidity sensor (GDX-CO2, Vernier Software & Technology, Oregon, USA).

Hyperspectral images of the bruised apples were acquired at seven time points after the damage, which were on 0 d (within 1 h after the impact), 1 d, 4 d, 8 d, 11 d, 15 d, and 18 d, so that storage day served as a source of temporal variability throughout the 18 d period. From 1 d onward, five apples per impact level were removed from cold storage at each time point, tempered at room temperature for 3 h, and then used for hyperspectral imaging followed by cross-sectional bruise area measurement. The number of apples used from each impact level and storage day is listed in **Table 1**, where some groups contained four apples because apples found with bruises that had not been induced as part of the experiment were excluded.

**Table 1:** Number of apples used for the acquisition of hyperspectral images and destructive measurement from each impact level and storage day.

Storage day	Level 1	Level 2	Level 3	Total
0d (imaging only)	29	30	28	87
1 d	5	5	5	15
4 d	5	5	5	15
8 d	5	5	4	14
11 d	5	5	5	15
15 d	4	5	4	13
18 d	5	5	5	15
Total	29	30	28	87

### 2.2 Acquisition and preprocessing of the hyperspectral images

#### 2.2.1 Image acquisition system

Hyperspectral images of the bruised apples were acquired with a line-scan hyperspectral sensor (FX10e, Spectral Imaging Ltd., Oulu, Finland) covering 400–1,000 nm with 224 spectral bands, by translating samples using a motorized conveyor, and each scan line formed 1,024 spatial pixels. Illumination was provided by two bar-type light sources mounted on both sides of the scan line and tilted at approximately 47°from the conveyor plane, each including three 100 W tungsten-halogen lamps (JCR 12V100W15H, Koninklijke Philips N.V., Amsterdam, Netherlands).

Six hyperspectral images per apple were acquired by rotating the fruit about its stem–calyx axis, so that bruises on the same sample were imaged at diverse radial positions on the fruit surface. For each hemisphere, one front view and two oblique views were acquired. The front view was used as the reference orientation, and the two oblique views were obtained by rotating the fruit by hand about its stem–calyx axis to approximately −30° and +30° relative to this orientation. Each cube was processed independently. Rotation was used to obtain images of the same bruise from different viewing directions rather than to image predefined positions on the fruit surface. Precise control and reproduction of the rotation angles were therefore unnecessary for this acquisition. During the bruise induction, bruises were mistakenly generated on only one side of some apples, resulting in only three images for these apples. In addition, some images with acquisition errors were removed before the analysis.

#### 2.2.2 Preprocessing of hyperspectral images

For each measurement session, white and dark reference frames were acquired and used for the reflectance calibration. A calibration plate with 95% reflectance (SG 3151, SphereOptics GmbH, Herrsching, Germany) was used as the white reference, and the dark frame was acquired by covering the sensor to block light (Nturambirwe et al., 2021). Reflectance was calculated for each wavelength according to Equation 2:

(2) $$R\left(\lambda \right)=\frac{I_{\text{raw}}\left(\lambda \right)-I_{\text{dark}}\left(\lambda \right)}{I_{\text{white}}\left(\lambda \right)-I_{\text{dark}}\left(\lambda \right)}$$

where *I*_raw_, *I*_white_, and *I*_dark_ are the digital numbers acquired from the sample, white reference, and dark frame, respectively, at wavelength *λ*.

Background pixels were removed by thresholding the per-pixel spectral standard deviation calculated across all wavelengths. Each apple region was then cropped into a reflectance cube of 500 × 500 pixels centered on the resulting apple mask, with pixels outside the apple set to zero.

#### 2.2.3 Bruise mask annotation

Reference bruise masks and bounding boxes were obtained by a two-stage workflow of manual box annotation followed by automated segmentation within each box (**Supplementary Figure S1**), because whole-image spectral transforms such as PCA and band-ratio images (Baranowski et al., 2013; Nturambirwe et al., 2021; Tian et al., 2023) did not robustly isolate bruises across the surface positions and impact levels in the dataset.

For the manual annotation, principal component (PC) score images were obtained by applying PCA to the 750–900 nm spectrum of each pixel, and a custom graphical interface (**Supplementary Figure S1**) displayed the PC1, PC2, and PC3 images. On the image in which bruise contrast was most pronounced, a loose rectangle enclosing each visible bruise with a surrounding margin was drawn in the 500 × 500 image coordinate system.

For the automated segmentation, glare pixels whose mean reflectance exceeded 0.95 were removed, and PCA was applied to the 750–900 nm reflectance of the remaining pixels inside each box. A normalized difference index (NDI) map was also calculated from the reflectance images at 720 and 800 nm (Tian et al., 2023), according to Equation 3:

(3) $$\text{NDI}=\frac{R\left(720\;\text{nm}\right)-R\left(800\;\text{nm}\right)}{R\left(720\;\text{nm}\right)+R\left(800\;\text{nm}\right)}$$

The three PC score maps and the NDI map were each binarized by Otsu thresholding and refined by hole filling, removal of small regions, and Gaussian smoothing of the boundary. The candidate mask that most clearly separated the bruise from the surrounding sound tissue was retained as the reference mask, and the reference bounding box of the instance was assigned accordingly.

Each bruise instance was additionally assigned a radial location category in each view (**Supplementary Figure S2**). The normalized radial position (*r*) was calculated by dividing the distance from the apple center to the bruise centroid by the distance from the center to the apple boundary along the same radial line. Instances were categorized as R1 ( 0≤r<1/3), R2 ( 1/3≤r<2/3), or R3 ( 2/3≤r≤1), corresponding to the central, intermediate, and peripheral zones of the visible apple surface. Because six views were acquired per apple, the same physical bruise could be assigned to different categories across views.

### 2.3 Quantification of bruise damage severity

#### 2.3.1 Indicators for bruise damage severity

As indicators for the damage severity, the level of impact applied for the damage and the level of internal browning observed in the cross-sectional image at the center of the bruise were used. Tissue damage associated with oxidative browning was quantified by the cross-sectional area of browned tissue at the center of the bruise, along with the maximum depth of the bruise from the peel. These measurements were obtained from one bruise region on each face of the apple.

#### 2.3.2 Measurement of the cross-sectional bruise area

To measure the cross-sectional area of the bruise represented by oxidative browning, RGB images of cut apple slices were acquired using a digital camera (*α* 6000, Sony Corp., Tokyo, Japan) mounted above the sample stage with LED lights (**Figure 2A**). The RGB values were calibrated on each experiment day using a 24-patch ColorChecker (X-Rite, Michigan, USA) and a 3 × 3 color correction matrix in the sRGB color space (**Figure 2B**). After the hyperspectral imaging at each time point, each apple was cut along a plane passing through the center of the bruise and perpendicular to the fruit surface, and a 10 mm thick slice was made from this cross-section for the imaging (**Figure 2C**).

**Figure 2:**
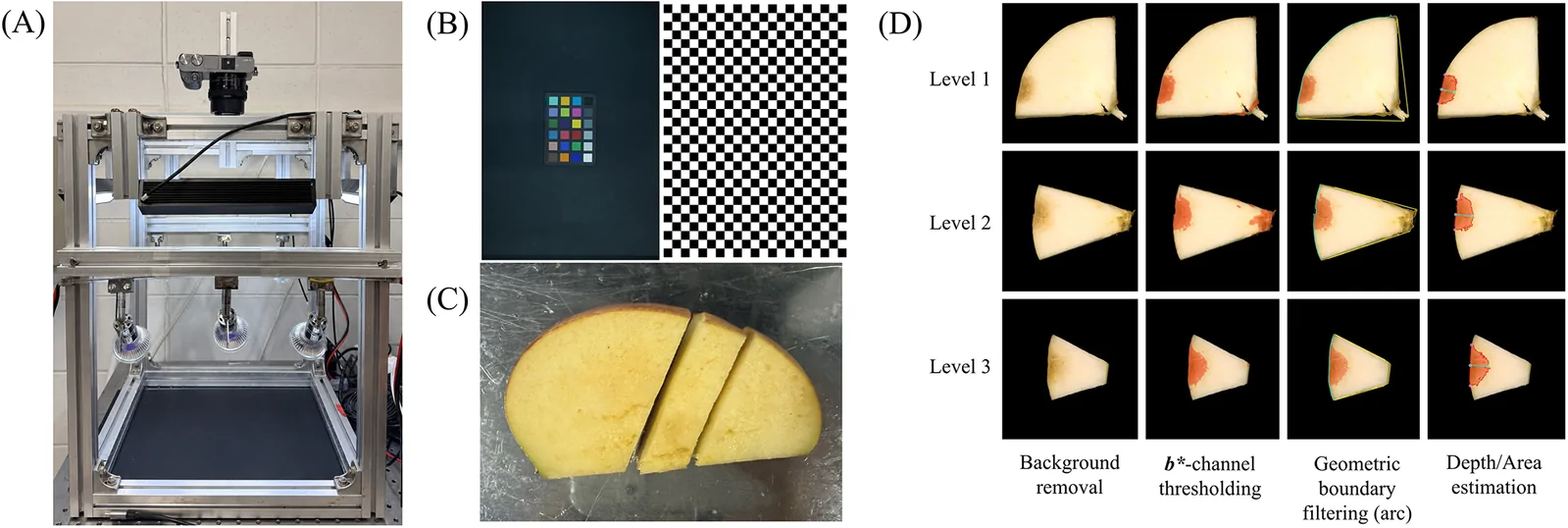
Workflow for cross-sectional bruise area measurement from RGB images of the cut apple surface. **(A)** Image acquisition system, **(B)** calibration targets, **(C)** a slice of the bruised tissue with 10 mm thickness, and **(D)** segmentation process of the browned area.

The browned tissue region was segmented from each calibrated image through the four steps illustrated in **Figure 2D**. Background pixels outside the slice were removed, and candidate browned pixels were defined by thresholding the 8-bit b^*^ channel of the CIE L^*^a^*^b^*^ color space at 165. Because sound tissue with a similar brown color, such as the tissue surrounding the seeds, was also captured by this color threshold, only the candidate regions adjacent to the peel, beneath which bruises formed, were retained. The peel arc was identified on the convex hull of the slice boundary as the edge remaining after the two longest straight segments were assigned to the cut surfaces. The pixel count of the retained region was converted into the cross-sectional bruise area in mm^2^ using a calibration factor of 0.0402 mm/px determined from a checkerboard with a square side length of 10 mm (**Figure 2B**), and the maximum inward penetration of the region from the peel arc was recorded as the bruise depth in mm. Bruise depth and cross-sectional bruise area were compared among the three impact levels at each storage day by one-way analysis of variance followed by Tukey’s honestly significant difference test at *p* < 0.05.

### 2.4 Deep spectral-spatial models for bruise detection and internal damage estimation

#### 2.4.1 Dataset partitioning

A total of 870 hyperspectral cubes with 1,676 annotated bruise instances were partitioned into train, validation, and test subsets at a 3:1:1 ratio, and the split was shared across the bruise detection, impact level classification, and cross-sectional bruise area regression tasks. The partitioning was conducted at the apple level so that all six views of an apple were assigned to the same subset, with stratified random sampling over the (impact level × storage day) groups defined in **Table 1**. The resulting subsets contained 51, 18, and 18 apples, corresponding to 513, 185, and 172 image cubes with 987, 356, and 333 reference bruise masks for training, validation, and testing, respectively. For the area regression, where the destructive measurement was performed on a single bruise instance from each side of an apple, 279, 104, and 109 instances were used. The entire partitioning is listed in **Supplementary Table S1**.

#### 2.4.2 Bruise detection

Bruise detection was formulated as pixel-wise prediction of a 2D binary mask from each hyperspectral reflectance cube, and four 3D U-Net variants using 3D convolutions for spectral–spatial feature extraction were trained and evaluated (**Figure 3**). The base 3D U-Net followed the encoder–decoder topology of the original U-Net and its 3D extension (Ronneberger et al., 2015; Çiçek et al., 2016), using a four-level 3D convolutional encoder with 12 base channels doubled at each level to 96, a symmetric 3D decoder, and skip connections between corresponding levels, with the spectral dimension collapsed only after the final decoder level to produce the 2D mask.

**Figure 3:**
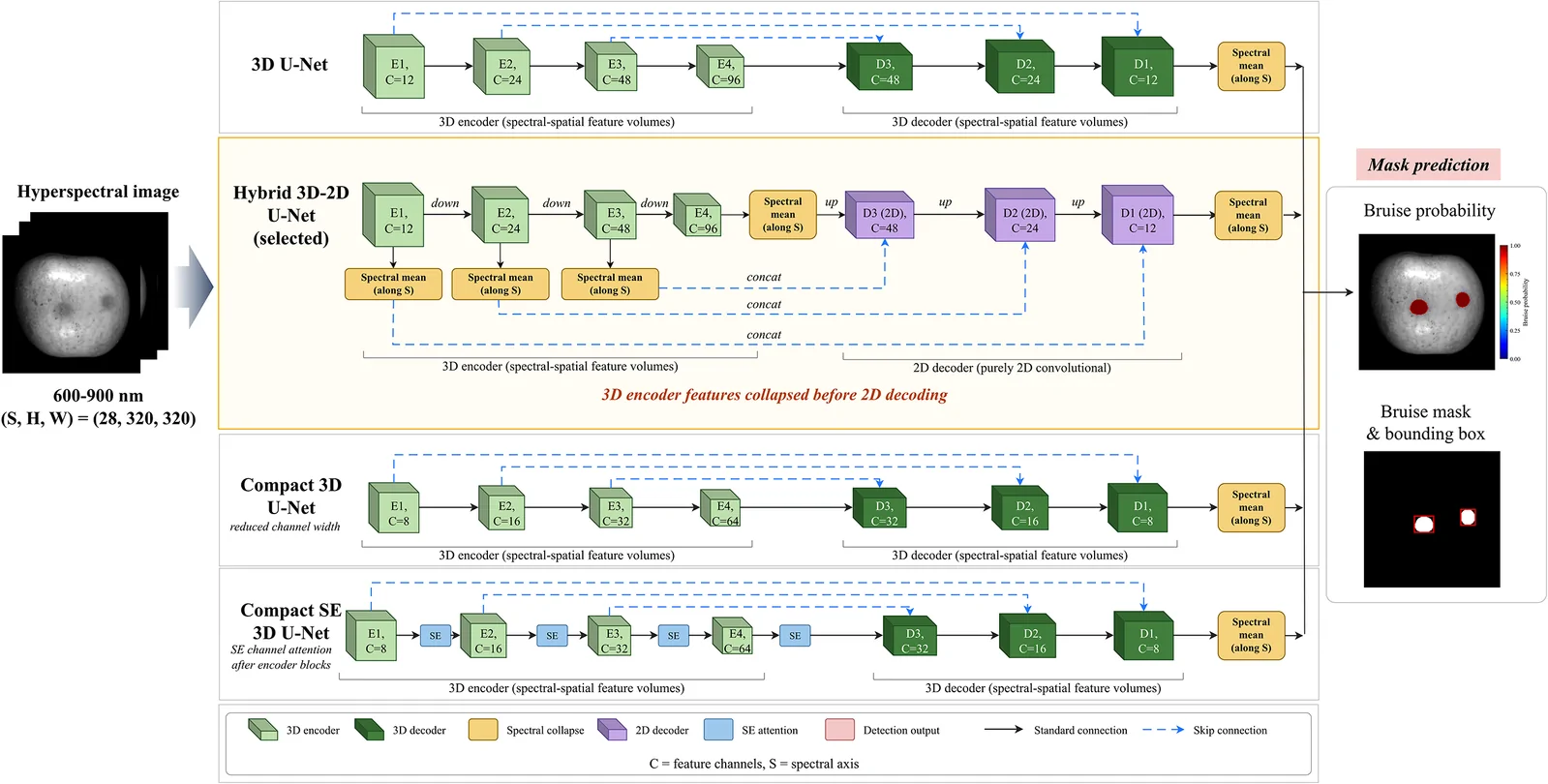
Architectures of the four 3D U-Net variants for bruise detection, which were 3D U-Net, Hybrid 3D–2D U-Net, Compact 3D U-Net, and Compact SE 3D U-Net (top to bottom).

Because the target mask was 2D, a Hybrid 3D–2D U-Net in which mask reconstruction was performed using 2D convolutions was evaluated. This model used the same four-level 3D encoder and base channel width as the base 3D U-Net, and the bottleneck feature and the encoder feature at each skip level were reduced along the spectral dimension by mean pooling and passed to the corresponding levels of a 2D U-Net decoder, following the general 3D-to-2D feature hierarchy of HybridSN (Roy et al., 2020).

Two additional variants were derived to determine whether model capacity could be reduced while retaining 3D decoding. The Compact 3D U-Net reduced the base channel width from 12 to 8 while retaining the 3D U-Net topology, and the Compact squeeze-and-excitation (SE) 3D U-Net additionally applied SE blocks to the encoder feature maps (Hu et al., 2018).

All four detectors received hyperspectral reflectance cubes of 600–900 nm, which covered the reported bruise-related ranges from approximately 680 nm into the near-infrared (Baranowski et al., 2013). The spectral axis was binned by a factor of four into 28 bands, and the two spatial axes were resized to 320 × 320 pixels, resulting in a model input of 28 × 320 × 320 corresponding to spectral bands, image height, and image width, respectively.

All four detectors were trained with the same mask loss in Equation 4:

(4) $$L_{\text{mask}}=L_{\text{BCE}}+L_{\text{Dice}}$$

where ${ℒ}_{\text{BCE}}$ is the pixel-wise binary cross-entropy with logits and ${ℒ}_{\text{Dice}}$ is the Dice loss computed from the predicted mask probability map and the reference bruise mask. The models were optimized with AdamW for up to 100 epochs. The learning rate was halved after three consecutive epochs without improvement in validation mask Dice, with a lower bound of 1 × 10^−6^, and training was stopped after 10 epochs without improvement.

As baseline detectors, YOLO11s and YOLO11s-seg were trained under the same apple-level data partition using three-channel images derived from the hyperspectral data, following previous studies that applied YOLO-based models to bruise detection in apples (Pang et al., 2022; Zhang et al., 2024). The input stacked the PC2 and PC3 score maps from a per-image PCA over 750–900 nm with the NDI image calculated from the 720 and 800 nm bands, reproducing the transforms for contrast enhancement used for mask annotation (*Section 2.2.3*) and in previous studies (Baranowski et al., 2013; Nturambirwe et al., 2021; Tian et al., 2023). The PC1 image was omitted because it mainly captured the spherical brightness gradient, whereas PC2, PC3, and NDI emphasized the contrast between bruised and sound tissue (Huang et al., 2015; Tian et al., 2023).

The influence of the input representation was further evaluated within YOLO11s and YOLO11s-seg using the same 28 spectral bands within 600 to 900 nm used by the U-Net variants. The first convolution was adapted to receive 28 channels. The remaining architecture was retained, and compatible pretrained weights were transferred. The original data partition and task-specific optimizer, learning rate schedule, augmentation settings, batch size of 16, and early stopping patience of 20 epochs were also retained. The models were trained for up to 150 epochs, and checkpoints were selected using validation performance.

A support vector machine (SVM) with a radial basis function kernel was evaluated as a conventional baseline for bruise detection, adapting the pixel classification approach of Baek et al. (2022). The model was trained on bruised and sound pixel spectra using the same 28 spectral bands and data partition as the proposed detector, and selected using validation performance. It was then applied to pixels in the test images, and the predicted bruise pixels were grouped into regions to obtain masks and bounding boxes.

#### 2.4.3 Impact level classification

The impact level assigned to each apple was used as the target of a three-class classification task. Three types of input feature sets were evaluated by training a separate classifier for each set, which were (1) features extracted from the pretrained Hybrid 3D–2D U-Net encoder, (2) explicit spectral–spatial statistics calculated from the bruise region, and (3) the mean reflectance spectrum of the bruise region. This comparison evaluated whether incorporating spatial information improved the assessment of damage severity relative to previous approaches based on one-dimensional spectra (Tan et al., 2018; Nturambirwe et al., 2020, 2021; Tang et al., 2020).

In the encoder representation, the pretrained Hybrid 3D–2D U-Net was reused as a spectral–spatial feature extractor with a classification head trained on top of it. The activations of the third and fourth encoder stages were passed through separate convolutional branches, spatially pooled, and concatenated for classification by two fully connected layers.

In the explicit spatial representation, five radial zones were defined by the relative pixel distance from the bruise centroid to the mask boundary, which were inner (0%–33%), middle (33%–67%), outer (67%–100%), near (100%–110%), and far (110%–120%). The mean and standard deviation spectra of each zone and the contrast spectra between the inside and surrounding zones were computed over 600–900 nm, standardized, and used to fit a ridge classifier.

In the one-dimensional representation, the mean reflectance spectrum within each bruise instance was classified by partial least squares discriminant analysis (PLS-DA) and an SVM with a radial basis function kernel. Preprocessing of the spectra was compared among raw reflectance, min–max normalization, maximum normalization, standard normal variate (SNV), and multiplicative scatter correction (MSC), the number of PLS-DA latent variables was searched over 1–20 components, and the SVM regularization constant was searched over C∈{1,10,100,1000} at γ=scale, all selected by the validation macro-averaged *F*1 score. As neural network baselines, a one-dimensional convolutional neural network (1D-CNN) and a multilayer perceptron using partial least squares scores (PLS-MLP) were evaluated. The 1D-CNN retained the convolutional and Inception structure and residual connection of SpectralCNN (Gai et al., 2022), with the input configuration adjusted for the 28-band spectra and the output layer configured for three-class impact level classification. The PLS-MLP consisted of two fully connected layers and used scores from the 19 latent variables of the selected PLS-DA configuration. The first fully connected layer width was searched over {8,16,24,32} for both models. Model configurations and training epochs were selected by validation macro-averaged *F*1. This baseline procedure was reused in its regression form for the cross-sectional bruise area estimation.

#### 2.4.4 Cross-sectional bruise area regression

The cross-sectional bruise area measured in *Section 2.3.2* was used as the regression target, with the aim of estimating the extent of subsurface browning from the surface hyperspectral image. As in the impact level classification, spectral–spatial features of the bruised tissue were extracted from the pretrained encoder. The third-stage encoder activations, spanning 48 channels by 14 spectral slices, were summarized by the mean activation within the inner, middle, and outer zones of the bruise mask (*Section 2.4.3*) for each channel and spectral slice, and by the sum of the activation over the whole mask. The normalized radial position of the bruise centroid (*Section 2.2.3*) and the three-class probabilities of the impact level classifier (*Section 2.4.3*) were appended to reflect the bruise location on the curved surface and the impact severity.

The encoder features were ranked by the absolute Pearson correlation with the cross-sectional area on the training subset, and up to 20 features with the highest correlations were combined with the appended position and probability features and fitted by ridge regression after standardization. The number of retained features and the ridge regularization strength were selected by the validation *R*^2^, with the feature number bounded so that the ratio of training instances to fitted feature dimensions stayed at or above approximately 10, limiting overfitting (Jain et al., 2000).

As one-dimensional baselines, partial least squares regression (PLSR) and support vector regression (SVR) with a radial basis function kernel were trained, following the same preprocessing search and selection used for the impact level classification (*Section 2.4.3*), based on validation *R*^2^.

As neural network baselines for cross-sectional bruise area estimation, 1D-CNN and PLS-MLP were also evaluated using the same mean reflectance spectra as the PLSR and SVR baselines. The network structures described in *Section 2.4.3* were retained, with the output layers configured for area prediction. The PLS-MLP consisted of two fully connected layers and used scores from the 20 latent variables of the selected PLSR configuration. Model configurations and training epochs were selected by validation *R*^2^.

#### 2.4.5 Evaluation metrics

Bruise detection performance was reported at the pixel level using the Dice score and the intersection-over-union (IoU) of the predicted binary bruise mask with respect to the reference mask, defined in Equations 5 and 6:

(5) $$\text{Dice}=\frac{2\left|\hat{M}\cap M\right|}{\left|\hat{M}\right|+\left|M\right|}$$

(6) $$\text{IoU}=\frac{\left|\hat{M}\cap M\right|}{\left|\hat{M}\cup M\right|}$$

where $\hat{M}$ and *M* are the sets of pixels assigned to the bruise class in the predicted and reference masks, respectively, and |·| denotes the pixel count.

From the predicted masks, the detection performance was reported using the precision, recall, and *F*1 score of the predicted bounding boxes at an IoU threshold of 0.5 between the predicted and reference bounding boxes. Each predicted bounding box was obtained by enclosing the connected region of predicted bruise pixels with the smallest rectangle aligned with the image axes. The precision, recall, and *F*1 score were defined in Equations 7–9:

(7) $$\text{Precision}=\frac{\text{TP}}{\text{TP}+\text{FP}}$$

(8) $$\text{Recall}=\frac{\text{TP}}{\text{TP}+\text{FN}}$$

(9) $$F1=\frac{2\times \text{Precision}\times \text{Recall}}{\text{Precision}+\text{Recall}}$$

where TP, FP, and FN refer to the numbers of true positive, false positive, and false negative bounding boxes, respectively. The performance and speed of the models were also compared at batch size 16 under FP32 and FP16 settings, including model inference and post-processing.

For the impact level classification, the performance was reported using the macro-averaged *F*1 score and the confusion matrix on the test partition. The macro-averaged *F*1 score was defined according to Equation 10:

(10) $$F1_{\text{macro}}=\frac{1}{K}\sum_{k=1}^{K}F1_{k}$$

where *K* = 3 is the number of impact level classes and *F*1*_k_* is the per-class *F*1 score of class *k*, computed by treating class *k* as the positive class and the remaining classes as negative.

For the cross-sectional bruise area regression, the performance was reported using the coefficient of determination *R*^2^, the Pearson correlation coefficient *r*, and the mean absolute percentage error (MAPE) in percent, defined in Equations 11–13:

(11) $$R^{2}=1-\frac{\sum_{i}{\left(x_{i}-y_{i}\right)}^{2}}{\sum_{i}{\left(x_{i}-\bar{x}\right)}^{2}}$$

(12) $$\begin{array}{c} r=\frac{\sum_{i}\left(x_{i}-\bar{x}\right)\left(y_{i}-\bar{y}\right)}{\sqrt{\sum_{i}{\left(x_{i}-\bar{x}\right)}^{2}\sum_{i}{\left(y_{i}-\bar{y}\right)}^{2}}} \end{array}$$

(13) $$\begin{array}{c} MAPE\left(\%\right)=\frac{1}{N}\sum_{i}\left|\frac{x_{i}-y_{i}}{x_{i}}\right|\times 100 \end{array}$$

where *x_i_* and *y_i_* denote the *i*-th reference value and the corresponding prediction, $\bar{x}$ and $\bar{y}$ their means, and *N* the number of evaluation samples.

## 3 Results and discussion

### 3.1 Spectral characteristics of bruised apple tissue

Mean reflectance spectra from the reference bruise masks were compared across radial viewing position, impact level, and storage period. The dataset used for modeling contained 357 central (R1), 759 intermediate (R2), and 560 peripheral (R3) bruise instances, with counts by impact level provided in **Supplementary Table S2**. Counts were obtained for individual instances in each image view because each physical bruise appeared at different radial positions across the six views.

Because the apple surface was curved, peripheral bruises (R3) were observed under more oblique viewing geometry than central bruises (R1), and this geometric effect produced systematic brightness differences across the radial regions, as reported for the reflectance distortion caused by surface curvature in hyperspectral imaging (Delwiche et al., 2021; Li et al., 2025; Wang et al., 2026). Reflectance spectra of an identical bruise observed at the R1, R2, and R3 positions across the rotated views were therefore compared before and after sample-wise min–max normalization (**Figure 4**). Without normalization, reflectance magnitude decreased monotonically from R1 to R3 across the measured wavelength range (**Figure 4A**). After the min–max normalization for each sample, this regional variation was mostly removed (**Figure 4B**), indicating that the radial reflectance difference originated from the brightness effect, with the underlying spectral shape largely shared across the radial regions. The group mean spectra over all bruise instances in each radial category showed similar trends (**Supplementary Figure S3**). Recent studies addressed this geometric effect by acquiring the surface geometry with an additional 3D point cloud camera to adjust the reflectance of each pixel and improve bruise detection accuracy in the edge region (Wang et al., 2026). In this study, the positional brightness effect was suppressed by the sample-wise min–max normalization using the hyperspectral cube alone, which is advantageous for practical sorting environments because the correction requires no additional sensing hardware or alignment between separate sensors.

**Figure 4:**
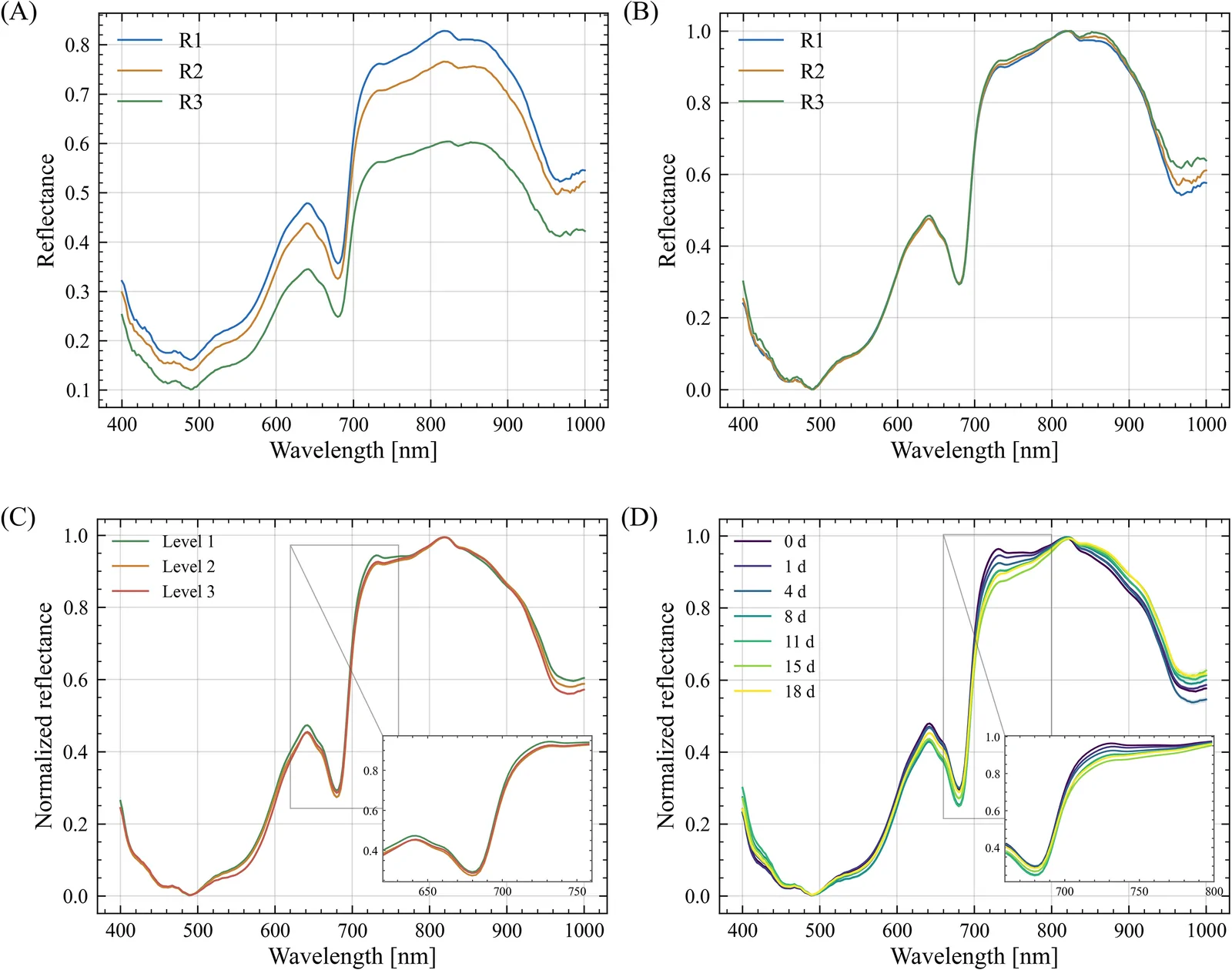
Reflectance spectra of bruised regions used to characterize spectral responses associated with bruise location, impact level, and storage period. **(A)** Spectra of an identical bruise observed at the R1, R2, and R3 positions across the rotated views, **(B)** the same spectra after sample-wise min–max normalization, **(C)** normalized spectra grouped by impact level, and **(D)** normalized spectra grouped by storage day.

After the suppression of the brightness effect, spectral responses across impact level and storage day were compared (**Figures 4C, D**). With higher impact level and longer storage after bruising, reduced reflectance around 700 nm was observed. This region overlaps the chlorophyll absorption band near 685 nm and the red-edge transition previously identified as effective for detecting bruises on apples (Mehl et al., 2004; Xing et al., 2005; ElMasry et al., 2008; Wang and ElMasry, 2010). More specifically, reflectance in the 650–734 nm band was reported to decline as browning developed in damaged apple tissue (Xu et al., 2025). The decreased reflectance was also associated with enzymatic browning, in which phenolic compounds released from the damaged cells were oxidized by polyphenol oxidase and accumulated as brown pigments that increased absorption in this region (Diels et al., 2017; Xu et al., 2025), and with altered light scattering caused by the disruption of the cell structure (Saeys et al., 2008; Zhang et al., 2021). A lower reflectance of bruised tissue at greater impact severity (Zhang et al., 2021) and spectral changes of bruised tissue with time after bruising (Baranowski et al., 2013) were also reported to be consistent with the ordering observed across the impact levels and storage days in **Figures 4C, D**.

### 3.2 Bruise severity in relation to impact level and storage period

Bruise depth and cross-sectional bruise area at each impact level and storage day are shown in **Figure 5**, with summary statistics and sample counts provided in **Supplementary Tables S3**, **S4**. Cross-sectional bruise area increased with impact level, with Level 3 consistently showing the largest browned tissue area and Level 1 the smallest (**Figure 5B**). The Tukey comparisons confirmed a significantly larger area for Level 3 than for Level 1 at every storage day, whereas differences involving Level 2 varied with storage day. Within each impact level, a clear monotonic change of the area with storage day was difficult to identify during 1 d to 18 d of the storage period. Bruise depth showed a similar ordering across impact levels with a smaller relative range (**Figure 5A**).

**Figure 5:**
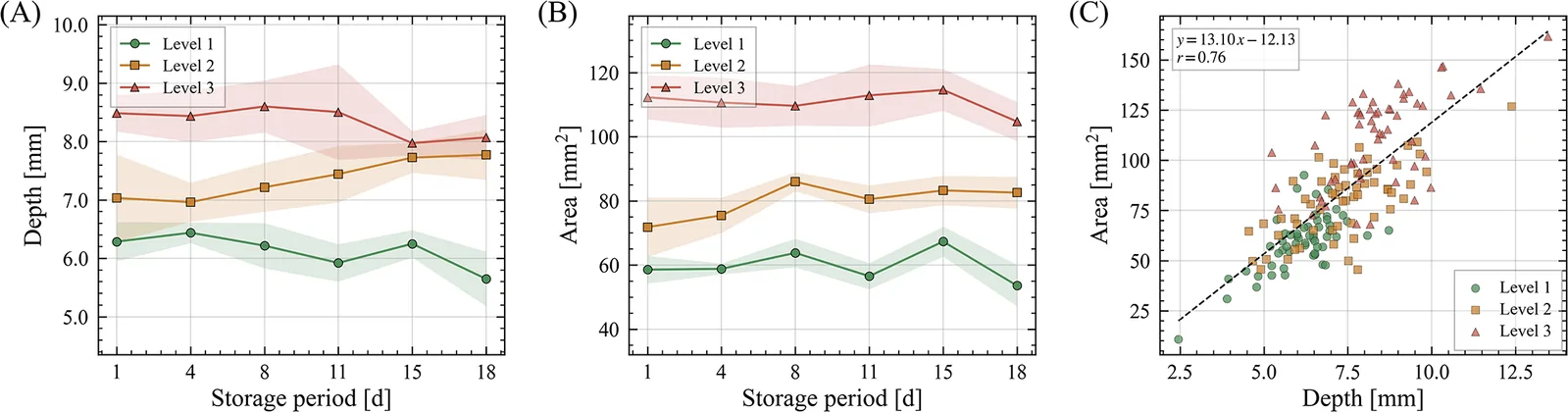
Bruise depth and cross-sectional bruise area across storage period and their relationship across individual bruises. **(A)** Bruise depth, **(B)** cross-sectional bruise area, and **(C)** depth–area scatter plot colored by impact level. Shaded bands indicate the standard error of the mean in **(A)** and **(B)**, and the dashed line indicates the linear fit in **(C)**.

The monotonic increase of the browned area with impact level was also reported in similar previous studies conducted using a drop test (Zhang et al., 2021), and higher drop energies with stresses beyond the bio-yield point of the flesh induced a permanently deformed region (Çelik et al., 2021). Browning in the damaged tissue developed through the oxidation of phenolic compounds released from the damaged cells (Diels et al., 2017; Çelik et al., 2021). Regarding the storage period, an increase in bruise area and volume was reported over a longer period, where the measurement was conducted at 30, 120, and 210 d (Çelik et al., 2021). Within the period of 18 d after the damage, a clear trend with storage day remained difficult to identify.

The relationship between bruise depth and cross-sectional bruise area across individual bruises is shown in **Figure 5C**. A positive linear relationship was observed between the two measurements (Pearson *r* = 0.76), indicating that deeper bruises were generally associated with larger cross-sectional browning extents.

### 3.3 Model performances for bruise detection and damage estimation

#### 3.3.1 Comparison of the bruise detection models

Detection accuracy under FP32 and FP16 inference and the corresponding processing speeds for the four 3D U-Net family detectors and four YOLO baselines, with two using three transformed channels and the other two using 28 spectral bands, are summarized in **Table 2** and **Figure 6**. All four variants trained on the spectrally resolved cubes reached higher detection accuracy than the YOLO baselines using three reduced channels, and the Hybrid 3D–2D U-Net was the most accurate and the fastest among the four variants.

**Table 2:** Accuracy under FP32 inference and processing speeds under FP32 and FP16 inference of the bruise detection models.

Architecture	Parameters (M)	Mask Dice	Box *F*1 (@IoU 0.5)	FPS (FP32)	FPS (FP16)
3D U-Net	1.40	0.8417	0.9657	15.38	31.85
**Hybrid 3D–2D U-Net**	**1.18**	**0.8607**	**0.9806**	**43.35**	**87.63**
Compact 3D U-Net	0.62	0.8360	0.9655	23.02	39.10
Compact SE 3D U-Net	0.63	0.8476	0.9776	22.59	38.42
YOLO11s (28 ch)	9.42	–	0.8963	431.50	720.53
YOLO11s-seg (28 ch)	10.07	0.7654	0.9141	299.69	440.80
YOLO11s (3 ch)	9.41	–	0.9141	407.17	718.98
YOLO11s-seg (3 ch)	10.07	0.6562	0.8455	331.92	582.10

The bold values indicate the results of the best-performing model.

**Figure 6:**
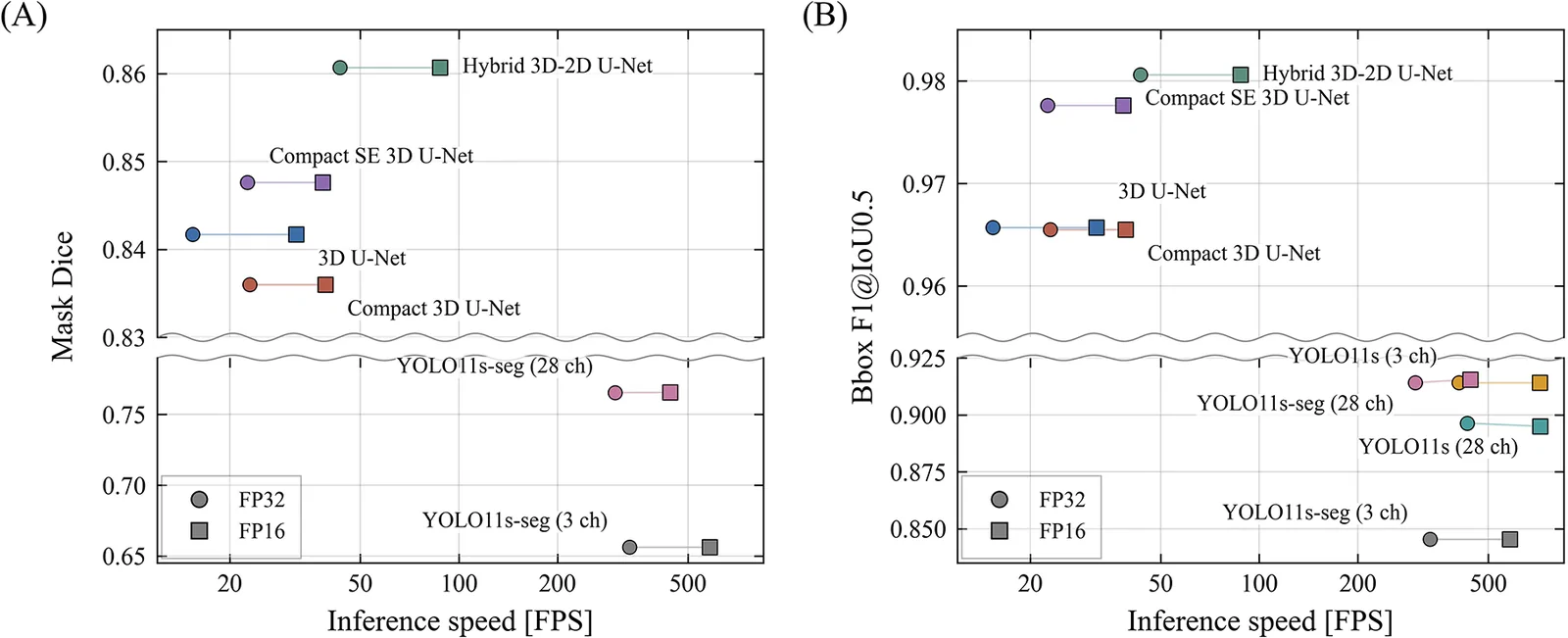
Detection accuracy of the bruise detection models with respect to inference speed. **(A)** Mask Dice against frames per second (FPS) on a logarithmic axis for the models that predicted masks. **(B)** Bounding box F1 at IoU 0.5 against FPS on a logarithmic axis.

Among the variants, replacing the 3D decoder with the 2D decoder improved both accuracy and speed. The Hybrid 3D–2D U-Net produced a higher mask Dice (0.8607) and bounding box *F*1 score (0.9806) than the base 3D U-Net (mask Dice: 0.8417, bounding box *F*1: 0.9657), with the inference speed approximately three times faster under FP32 (43.35 against 15.38 FPS). This result indicated that collapsing the spectral dimension after encoding was sufficient for reconstructing the 2D mask. The two compact variants, which were designed with narrower channels to examine whether model capacity could be reduced while retaining the 3D decoder, achieved accuracy comparable to the base model with roughly half the parameters, and the SE blocks in the Compact SE 3D U-Net slightly improved both accuracy metrics over the Compact 3D U-Net. However, both variants remained slower and less accurate than the Hybrid 3D–2D U-Net. In addition, for the four U-Net variants, reducing the inference precision from FP32 to FP16 preserved the mask Dice and the bounding box *F*1 score to four decimal places while roughly doubling the processing speed, indicating that FP16 inference was sufficient for bruise detection from the hyperspectral cubes in this framework.

The YOLO11s and YOLO11s-seg baselines using three transformed channels reached higher FPS but produced lower bounding box *F*1 scores (0.9141 and 0.8455, respectively) and a lower mask Dice (0.6562 for YOLO11s-seg), despite approximately eight times more parameters than the Hybrid 3D–2D U-Net (**Table 2**). Replacing the three transformed channels with 28 spectral bands at the same spatial resolution improved the bounding box *F*1 score of YOLO11s-seg from 0.8455 to 0.9141 and its mask Dice from 0.6562 to 0.7654. In contrast, the same input change slightly decreased the bounding box *F*1 score of YOLO11s from 0.9141 to 0.8963. For the additional 28-channel models, FP16 inference increased processing speed from 431.50 to 720.53 FPS for YOLO11s and from 299.69 to 440.80 FPS for YOLO11s-seg. Small changes in accuracy were observed. The bounding box *F*1 score changed from 0.8963 to 0.8950 for YOLO11s and from 0.9141 to 0.9155 for YOLO11s-seg. Mask Dice for YOLO11s-seg changed from 0.7654 to 0.7656. The 87.63 FPS of the selected model under FP16 was also comparable to the processing speeds of 68 and 130 FPS reported for YOLO-based bruise detection in apples (Pang et al., 2022; Zhang et al., 2024), indicating that the accuracy gain from the spectrally resolved input was obtained at a speed relevant to inline inspection.

Compared to deep learning approaches, the conventional SVM approach following the method of Baek et al. (2022) using the same 28 spectral bands showed the lowest detection performance. The SVM achieved a bounding box *F*1 score of 0.5057 and an instance Dice of 0.4326, compared with 0.9806 and 0.8607 for the Hybrid 3D–2D U-Net. Its bounding box precision and recall were 0.4811 and 0.5329, compared with 0.9763 and 0.9850 for the Hybrid 3D–2D U-Net. Both false detections and missed bruises therefore limited the SVM performance across the complete images. Each pixel was classified using only its spectrum without spatial information. For the same samples shown in **Figure 7**, false positives associated with surface noise and frequent false negatives at peripheral bruises were observed (**Supplementary Figure S4**). These results supported the effectiveness of the proposed framework relative to the evaluated conventional baseline under the same spectral range and data partition.

**Figure 7:**
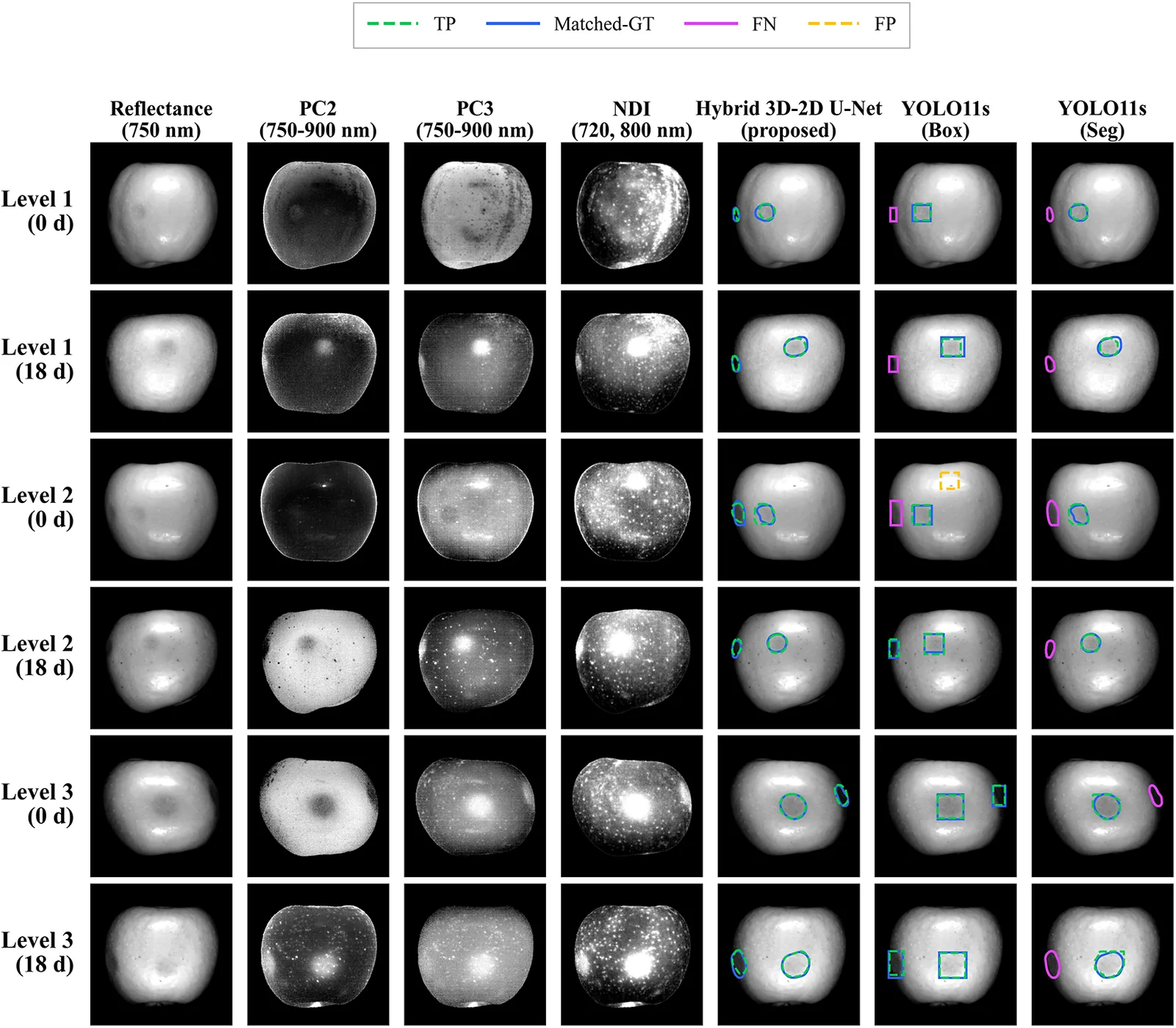
Examples of bruise detection results comparing the selected Hybrid 3D–2D U-Net with the two YOLO baselines using three transformed channels across impact level and storage day.

The wavelength ablation supported the selected 600 to 900 nm range as a compact input for bruise detection (**Supplementary Table S5**). Three additional Hybrid 3D–2D U-Net models were trained using the full 400 to 1,000 nm, 400–600 nm, and 900–1,000 nm ranges. The same spectral binning interval as the original 600–900 nm model yielded 56, 18, and 9 input bands, respectively, and the original data partition, preprocessing, training settings, and test evaluation procedure were retained. The selected range produced the highest instance Dice of 0.8607, followed by the full range with a Dice of 0.8431, the 900 to 1,000 nm range with a Dice of 0.7954, and the 400 to 600 nm range with a Dice of 0.7625. Its bounding box *F*1 score of 0.9806, obtained with half the input bands, was also comparable to 0.9805 for the full range. Thus, although both excluded regions contained useful information, extending the input to the full range provided no improvement in these detection metrics under the evaluated conditions.

#### 3.3.2 Stratified detection performance by bruise location, impact level, and storage period

In previous studies, bruise detection was evaluated under conditions such as bruises imaged with the bruised surface facing the camera (Gai et al., 2022), bruises produced at a single impact level (Zhang et al., 2024), or bruises imaged at a fixed time after impact (Keresztes et al., 2016). In this study, these three conditions were varied to include central, intermediate, and peripheral bruises, three impact levels, and seven storage periods from 0 to 18 d. Robustness referred to the detection accuracy maintained across these varied conditions.

The robustness of the detection was further examined by stratifying the bounding box *F*1 score of the Hybrid 3D–2D U-Net variant and the two YOLO baselines using 28 spectral bands by bruise location, impact level, and storage day (**Table 3**). Although the impact energy of an individual fruit cannot be known in practice, this stratified evaluation was performed to verify that the detection model performed stably on bruises with different levels of difficulty, such as mild bruises (Level 1), early bruises (0 d), and peripheral bruises (R3). In the evaluation stratified by location, the Hybrid 3D–2D U-Net produced higher *F*1 scores than both baselines in all three categories of the bruise location. Although the *F*1 score decreased from the central toward the peripheral region for every model, the decrease from R1 to R3 was much smaller for the Hybrid 3D–2D U-Net, indicating higher accuracy under the oblique viewing geometry of peripheral bruises. Similarly, across impact levels, the Hybrid 3D–2D U-Net remained more accurate than both baselines, indicating its robustness in detecting mild bruises. For bruises with different storage periods, the model outperformed the baselines at every storage period.

**Table 3:** Bounding box *F*1 score at an IoU threshold of 0.5 for the selected Hybrid 3D–2D U-Net and the YOLO11s and YOLO11s-seg baselines using 28 spectral bands, stratified by bruise location, impact level, and storage day.

Stratification factor	Group	Hybrid 3D–2D U-Net	YOLO11s (28 ch)	YOLO11s-seg (28 ch)
Bruise location	R1	**1.0000**	0.9940	0.9630
	R2	**0.9887**	0.9189	0.9249
	R3	**0.9869**	0.8125	0.8908
Impact level	Level 1	**0.9805**	0.8516	0.8907
	Level 2	**0.9560**	0.8824	0.8914
	Level 3	**1.0000**	0.9565	0.9565
Storage day	0 d	**0.9821**	0.8635	0.8881
	1 d	**0.9688**	0.8621	0.8727
	4 d	**0.9722**	0.8986	0.9275
	8 d	**1.0000**	0.9583	0.9787
	11 d	**0.9851**	0.9552	0.9412
	15 d	**1.0000**	0.9859	0.9589
	18 d	**0.9577**	0.8611	0.9167

The bold values indicate the results of the best-performing model.

Taken together, large gaps between the selected model and the baselines occurred for peripheral bruises (R3), the earliest bruises (0 d), and the mildest bruises (Level 1). In these groups, the selected model retained *F*1 scores of at least 0.98, while the baseline scores ranged from 0.81 to 0.89. Across all of the stratified groups, the *F*1 score of the selected model remained at or above 0.95, and this consistent accuracy under the varying bruise location, impact severity, and storage period indicated the robustness of the detection. Because 0 d corresponded to imaging within 1 h of impact, this result also supported the effectiveness of the selected model for detecting early bruises defined as bruises formed within 1 to 2 h (Sun et al., 2022).

The advantage of the model for the mildest bruises (Level 1) was further interpreted in relation to the impact energies used in previous studies. Bruising in apple flesh has been reported to begin at impact energies of approximately 0.03 to 0.05 J (Komarnicki et al., 2017), and earlier hyperspectral studies with Vis/NIR (400–1,000 nm) and short-wave infrared (SWIR, 1,000–2,500 nm) imaging induced bruises over a range of approximately 0.10 to 0.80 J (Baranowski et al., 2012; Keresztes et al., 2017). The three impact levels applied in this study (0.147 J for Level 1, 0.294 J for Level 2, and 0.539 J for Level 3) fell within this range, and Level 1 was approximately 48% lower than the lowest severity level (0.28 J) described under the same steel ball drop approach (Nturambirwe et al., 2020). The high *F*1 score retained at Level 1 therefore indicated that the high detection accuracy was obtained for a comparatively mild bruise. However, further study is required to determine the smallest impact at which a bruise remains detectable with the selected detector.

Representative detection examples across impact level and storage day are shown in **Figure 7**. In the first column, the reflectance image at 750 nm is presented as a representative wavelength at which the spectral difference between bruised and sound tissue was observed (*Section 3.1*), and in the following three columns, the PC2, PC3, and NDI maps that composed the three-channel input of the YOLO baselines are displayed. In the remaining three columns, the detection results of each model are presented. Because all of these panels were derived from the hyperspectral cube, the bruise contrast in **Figure 7** reflected the near-infrared response of the bruised tissue. The same response formed the contrast of the early bruises imaged at 0 d within 1 h after the impact, which are difficult to identify with conventional color imaging because the internal browning of the early bruise develops without a visible change on the peel (ElMasry et al., 2008; Li et al., 2025). The bruises near the fruit boundary (R3) were frequently missed by the YOLO baselines, and a false positive was also produced in the glare region of the Level 2 sample imaged at 0 d. These bruises were detected by the proposed approach combining 28 spectral bands with 3D convolutions in a U-Net framework, illustrating its effectiveness in cases where the YOLO baselines using three transformed channels failed to detect them.

The pronounced advantage in R3 samples addressed the reported difficulty of detecting bruises near the fruit periphery, where the brightness gradient of the curved surface weakens the contrast of bruised regions (*Section 3.1*). Recent studies compensated for this effect using explicit information on the 3D shape of the fruit, including radial grid correction, geometric correction adapted to fruit size, and point cloud geometry (Castro et al., 2023; Li et al., 2025; Wang et al., 2026). In this study, a more practical approach was attempted by combining the sample-wise min–max normalization using the hyperspectral cube alone with the detection model based on the 3D U-Net architecture, requiring no additional sensing hardware or alignment between separate sensors. The detection accuracy retained at R3 supported the feasibility of this approach for inspecting bruises across the curved fruit surface.

#### 3.3.3 Bruise severity prediction

As predicting bruise severity is more meaningful for practical quality assessment, bruise severity was predicted as categorical impact level and continuous cross-sectional bruise area, representing applied impact energy and the extent of internal browning, respectively. For impact level classification, the classification head trained on the Hybrid 3D–2D U-Net encoder features achieved a test macro-averaged *F*1 score of 0.83 (**Table 4**). In comparison, the ridge classifier using the mean and standard deviation spectra of the radial zones reached 0.63, and test macro *F*1 scores of 0.52 to 0.58 were obtained with PLS-DA and SVM, compared with 0.59 to 0.60 with the 1D-CNN and PLS-MLP models using the same mean reflectance spectra. Although a slightly higher score was obtained with the 1D-CNN (0.60) than with SVM (0.58), the highest score of 0.83 was obtained with the classifier using the pretrained encoder, suggesting that the learned representation retained information useful for impact level classification beyond the mean spectrum. Because instances from all seven storage periods were included, these values represented discrimination under temporal changes in bruise appearance. The advantage of the representation based on radial zones over the one-dimensional baselines indicated that spatial variation within the bruise carried information beyond the mean spectrum, while the further improvement obtained with the encoder suggested that it captured additional spectral–spatial patterns. This finding extended previous assessments of impact severity in apples that relied primarily on one-dimensional hyperspectral or near-infrared spectra (Tan et al., 2018; Nturambirwe et al., 2020, 2021; Tang et al., 2020).

**Table 4:** Impact level classification performance based on spectral-spatial features and spectral-only features.

Model	Input features	Preprocessing	Train *F*1	Val. *F*1	Test *F*1
**Hybrid 3D–2D U-Net encoder + classification head**	**600–900 nm cube**	**–**	**0.9791**	**0.8739**	**0.8345**
Ridge	Mean, standard deviation spectra from each radial zone	Standardization	0.7909	0.7244	0.6253
PLS-DA	Mean reflectance spectrum (1D)	SNV	0.6715	0.6551	0.5185
SVM	Mean reflectance spectrum (1D)	MSC	0.6886	0.5827	0.5846
1D-CNN (SpectralCNN)	Mean reflectance spectrum (1D)	Min–max	0.6946	0.6745	0.5973
PLS-MLP	Mean reflectance spectrum (1D)	SNV	0.7940	0.6767	0.5850

The bold values indicate the results of the best-performing model.

The confusion matrices showed that Level 1 and Level 3 were classified more consistently than Level 2 (**Figure 8**). In the test subset, most remaining errors were associated with Level 2 instances that were assigned to either adjacent impact level, whereas no Level 1 instance was assigned to Level 3 and no Level 3 instance was assigned to Level 1. Confusion among different impact levels was further investigated for each radial category of the bruises (**Supplementary Figure S5**). For bruises in R1, R2, and R3 regions, the test macro *F*1 scores were 0.88, 0.84, and 0.79, respectively. In all three regions, errors occurred between adjacent impact levels, with no misclassification between Level 1 and Level 3. These results suggested that reflectance differences caused by the radial position of the bruises were not primarily associated with the confusion of the impact levels of the bruises. This error pattern was consistent with the overlap of the cross-sectional bruise area of Level 2 with the adjacent levels observed across storage days (*Section 3.2*).

**Figure 8:**
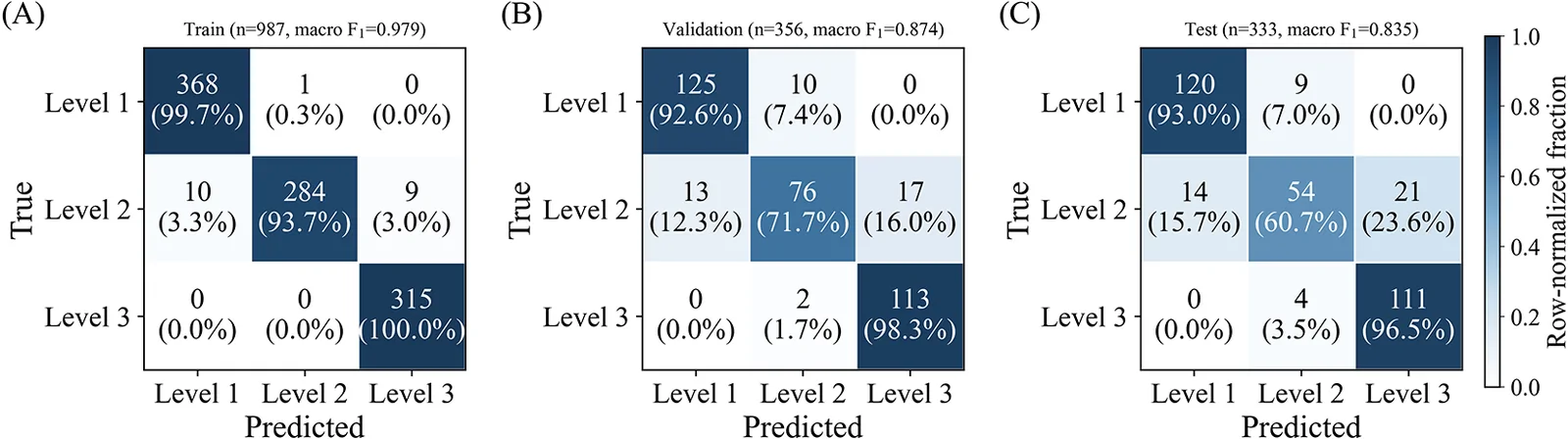
Confusion matrices of the impact level classifier with the Hybrid 3D–2D U-Net encoder on the **(A)** train, **(B)** validation, and **(C)** test subsets.

To present feature-level interpretation of the impact level classifier, the final 128 features immediately before the classification output layer were extracted from all test bruise instances and visualized using t-distributed stochastic neighbor embedding (t-SNE) (Maaten and Hinton, 2008) in **Supplementary Figure S6**. These features combined encoder information with reflectance from the bruise region. Level 1 and Level 3 were largely distributed in separate regions, while Level 2 partly overlapped with both adjacent levels. This distribution was consistent with the classification errors in **Figure 8** and suggested that information related to impact level was retained in the final learned representation. The visualization described the distribution of the combined classification features across bruise instances, complementing the spatial activation of an individual encoder feature associated with cross-sectional bruise area discussed below (**Figure 9**). The t-SNE distribution was interpreted qualitatively and did not directly represent the classification boundaries. Accordingly, the assessment of the damage severity on a continuous scale was addressed by the estimation of the cross-sectional bruise area.

**Figure 9:**
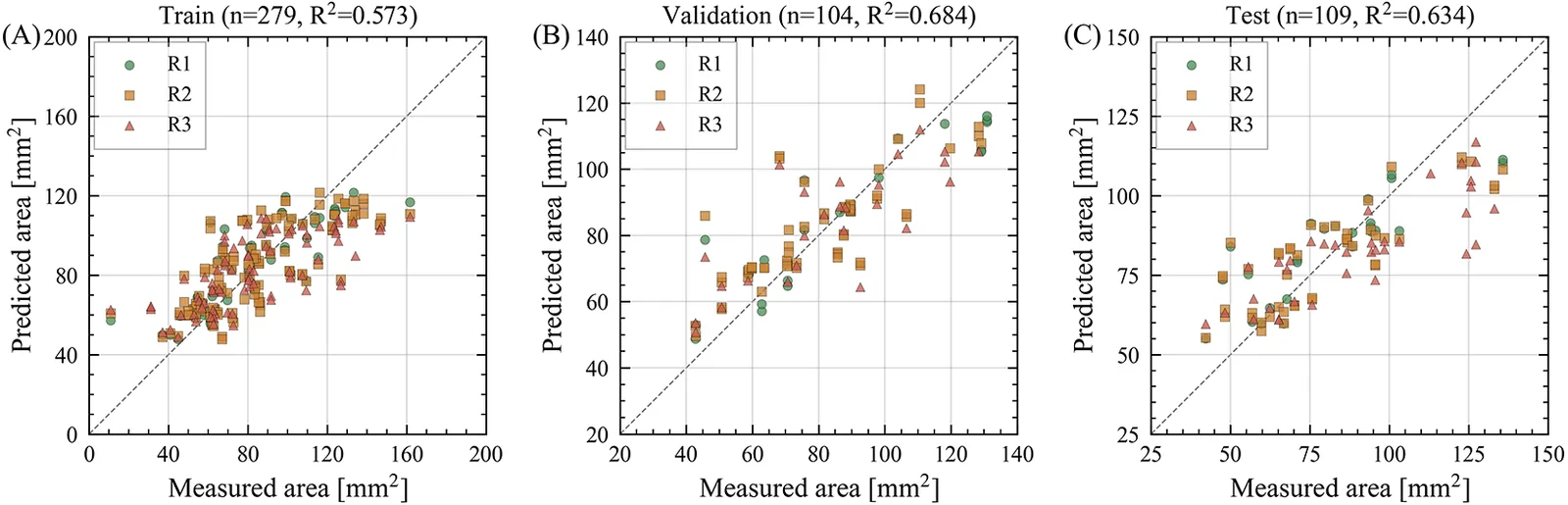
Scatter plots of predicted and reference cross-sectional bruise area for the **(A)** train, **(B)** validation, and **(C)** test subsets. The dashed line indicates the 1:1 relationship.

For cross-sectional bruise area estimation, ridge regression using the spatially resolved encoder features together with the radial position and the impact level probabilities outperformed the models based on the mean reflectance spectrum, reaching a test *R*^2^ of 0.63, Pearson *r* of 0.82, and MAPE of 15.40% (**Table 5**). The training *R*^2^ of 0.57 was lower than the validation and test values because strong ridge regularization was selected on the validation subset, and reducing the regularization strength increased the training *R*^2^ but decreased the validation *R*^2^ over the examined range. In comparison, Pearson *r* values of 0.58 and 0.65 were obtained with 1D-CNN and PLS-MLP using the mean reflectance spectrum, respectively, exceeding that of SVR (0.51) but remaining below that of PLSR (0.67). The higher correlations obtained with PLSR and PLS-MLP than with 1D-CNN and SVR suggested that the PLS representation effectively captured information related to bruise area in the mean spectra. The similar correlations of PLSR and PLS-MLP further suggested that nonlinear modeling of the PLS scores provided limited additional benefit under the present conditions. The improvement over PLSR, SVR, 1D-CNN, and PLS-MLP was consistent with the advantage of the spatially resolved features observed in the impact level classification. Accordingly, the scatter plots in **Figure 9** reflected the strong linear association and the moderate explained variance of the area prediction across the three data subsets. Nevertheless, these results supported the sensitivity of the surface measurement to the changes beneath the peel. The impact level and the cross-sectional bruise area both represented the damage that developed in the subsurface tissue, and the estimation results from the hyperspectral images reached a test macro *F*1 score of 0.83 and the test *R*^2^ of 0.63 (**Table 4**, **5**). These results indicated that the hyperspectral system used in this study was sensitive enough to capture spectral changes of the damaged subsurface tissues beneath the peel. Damage extending beyond the reported penetration depth of approximately 1.8 mm in apple tissue was estimated indirectly from the spectral responses (Zhou et al., 2023).

**Table 5:** Cross-sectional bruise area regression performance based on spectral-spatial features and spectral-only features.

Model	Input features	Preprocessing	Train			Validation			Test		
			*R* ^2^	*r*	MAPE (%)	*R* ^2^	*r*	MAPE (%)	*R* ^2^	*r*	MAPE (%)
**Ridge**	Encoder features (18)^a^ **Radial position (1)** **Impact level probabilities (3)**	**Standardization**	**0.5725**	**0.7610**	**23.29**	**0.6842**	**0.8352**	**14.35**	**0.6337**	**0.8246**	**15.40**
PLSR	Mean reflectance spectrum (1D)	SNV	0.5012	0.7080	24.00	0.2663	0.5956	20.27	0.4378	0.6696	27.61
SVR	Mean reflectance spectrum (1D)	MSC	0.1708	0.4589	28.32	0.0432	0.3315	22.02	0.2102	0.5062	26.53
1D-CNN	Mean reflectance spectrum (1D)	Raw	0.1968	0.4453	30.63	0.1891	0.4555	22.31	0.3347	0.5847	26.00
PLS-MLP	Mean reflectance spectrum (1D)	SNV	0.5556	0.7519	21.59	0.2926	0.5639	19.03	0.4119	0.6483	26.96

^a^ Of the 18 selected encoder features, 11 were mean activations calculated within the inner, middle, and outer zones, and seven were activations summed over the entire bruise mask. Values in parentheses indicate the number of scalar input features.

The bold values indicate the results of the best-performing model.

**Figure 10:**
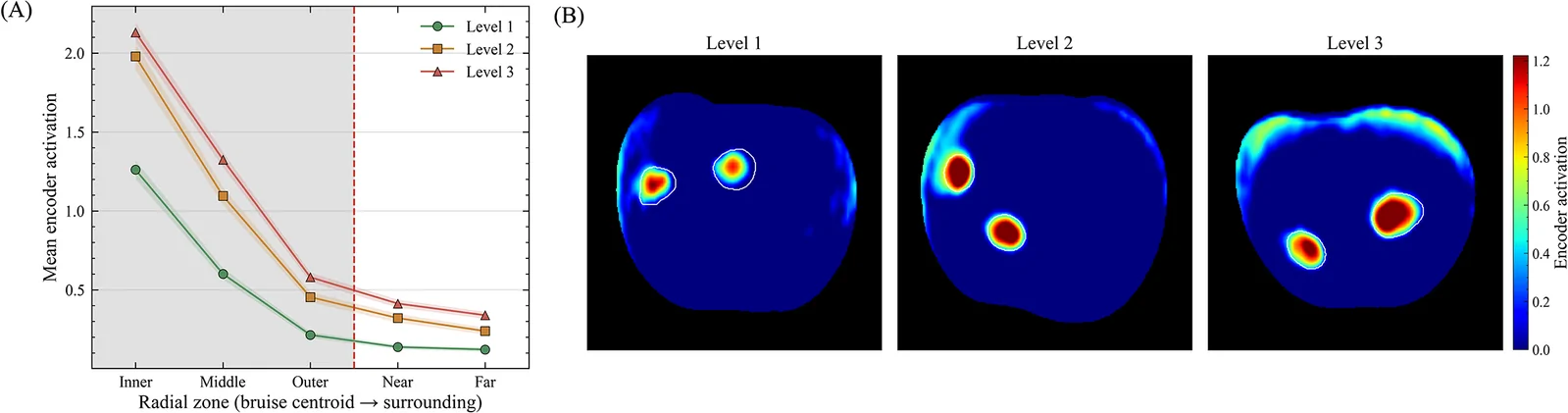
Encoder activation associated with the cross-sectional bruise area regression. **(A)** Mean activation across the five radial zones centered on the bruise, extending from the bruise centroid into the surrounding tissue, for each impact level, and **(B)** activation maps of the entire fruit for representative Level 1, Level 2, and Level 3 apples.

The encoder features selected for the area regression were distributed within 750–820 nm and were concentrated in spectral slices centered at 759 and 781 nm. The feature most strongly correlated with cross-sectional bruise area is shown in **Figure 10**. The activation of the encoder was concentrated in the inner and middle zones of the bruise and increased from Level 1 to Level 3, following the ordering of cross-sectional area (*Section 3.2*). Its band near 780 nm fell within the range reported as responsive to changes induced by bruising in apple tissue (ElMasry et al., 2008; Huang et al., 2015). Together with the distribution of the final classification features in **Supplementary Figure S6**, this spatial activation illustrated how the model distinguished bruises and their severity levels.

Previous hyperspectral studies of bruises in apples focused on detecting and visualizing the bruised region from the surface images (Xu et al., 2019; Zhang et al., 2021, 2023; Li et al., 2024). However, because the actual damage propagates beneath the peel (Sidhu et al., 2023), its internal extent has been quantified by destructive sectioning, X-ray computed tomography, or finite-element simulation (Diels et al., 2017; Wood et al., 2024; Xu et al., 2024). In this study, the hyperspectral assessment was extended from the detection to the quantitative estimation of this internal damage by regressing the cross-sectional area of the browned tissue. This extension was physically constrained by the shallow penetration of the reflectance measurement, which probes a subsurface layer of about 1.8 mm in intact apple tissue (Zhou et al., 2023). Because the bruises extended well beyond this depth, the status at the deeper part of the tissue was only indirectly measured, which explained the strong correlation and the moderate explained variance of the area prediction.

## 4 Conclusions

In this study, a deep spectral–spatial framework was developed for robust bruise detection and estimation of internal damage from Vis/NIR hyperspectral images of apples, and was evaluated using 1,676 bruise instances spanning three impact levels, central to peripheral surface locations, and seven storage periods up to 18 d. Among the four 3D U-Net variants receiving the spectrally resolved cubes, the Hybrid 3D–2D U-Net combining 3D spectral–spatial encoding with 2D mask reconstruction was the most accurate and the fastest, achieving a mask Dice of 0.86 and a bounding box *F*1 score of 0.98 at 87.63 FPS under FP16 inference with both accuracy metrics preserved. The model also outperformed the YOLO baselines using three reduced channels or the same 28 spectral bands. In particular, the advantage over the baselines was pronounced for the challenging cases, which were peripheral, early, and mild bruises, with *F*1 scores of at least 0.98 and no additional geometric sensing or correction hardware.

As a step further, hyperspectral bruise detection was extended to the estimation of internal damage by reusing the encoder features of the selected detector. From the encoder features trained for the bruise detection, a test macro-averaged *F*1 score of 0.83 was achieved for impact level classification. For internal cross-sectional bruise area, a target that had not previously been addressed through hyperspectral regression, a test *R*^2^ of 0.63 and Pearson *r* of 0.82 were achieved by combining the encoder features with the location and the impact level probabilities of the bruise. In both tasks, the spatially resolved features outperformed the models based solely on the mean reflectance spectrum, indicating that the spatially distributed information within the bruise region was effective for estimating the characteristics of the damage.

Taken together, it was demonstrated that robust bruise detection and quantitative estimation of internal damage were achievable through the proposed spectral–spatial framework. However, several limitations exist for the practical application of the model. Validation across additional cultivars, harvest seasons, storage conditions, and larger independent sample sets remains necessary to assess the generalizability of bruise detection and severity estimation. For inline application, manual reorientation needs to be replaced by automatic fruit rotation, with further evaluation of detection accuracy and speed during acquisition of the entire fruit surface. In addition, the bruises in this study were induced by dropping a steel ball, simplifying the bruise shapes to circles, so the detection of bruises with irregular shapes formed under diverse impact conditions remains a subject for further investigation before the inline application.

## Data Availability

The data supporting the findings of this study are available from the corresponding author upon reasonable request.
