# Supplementary material for: Deep spectral–spatial learning for robust bruise detection and internal damage estimation in apples using visible/near-infrared hyperspectral imaging
Source: Front Plant Sci. 2026 Oct 27;17:1967857. doi: 10.3389/fpls.2026.1967857 (PMC13747794; doi:10.3389/fpls.2026.1967857)
Supplement: Supplementary file 1 [file DataSheet1.pdf]

Supplementary Material

1 SUPPLEMENTARY FIGURES

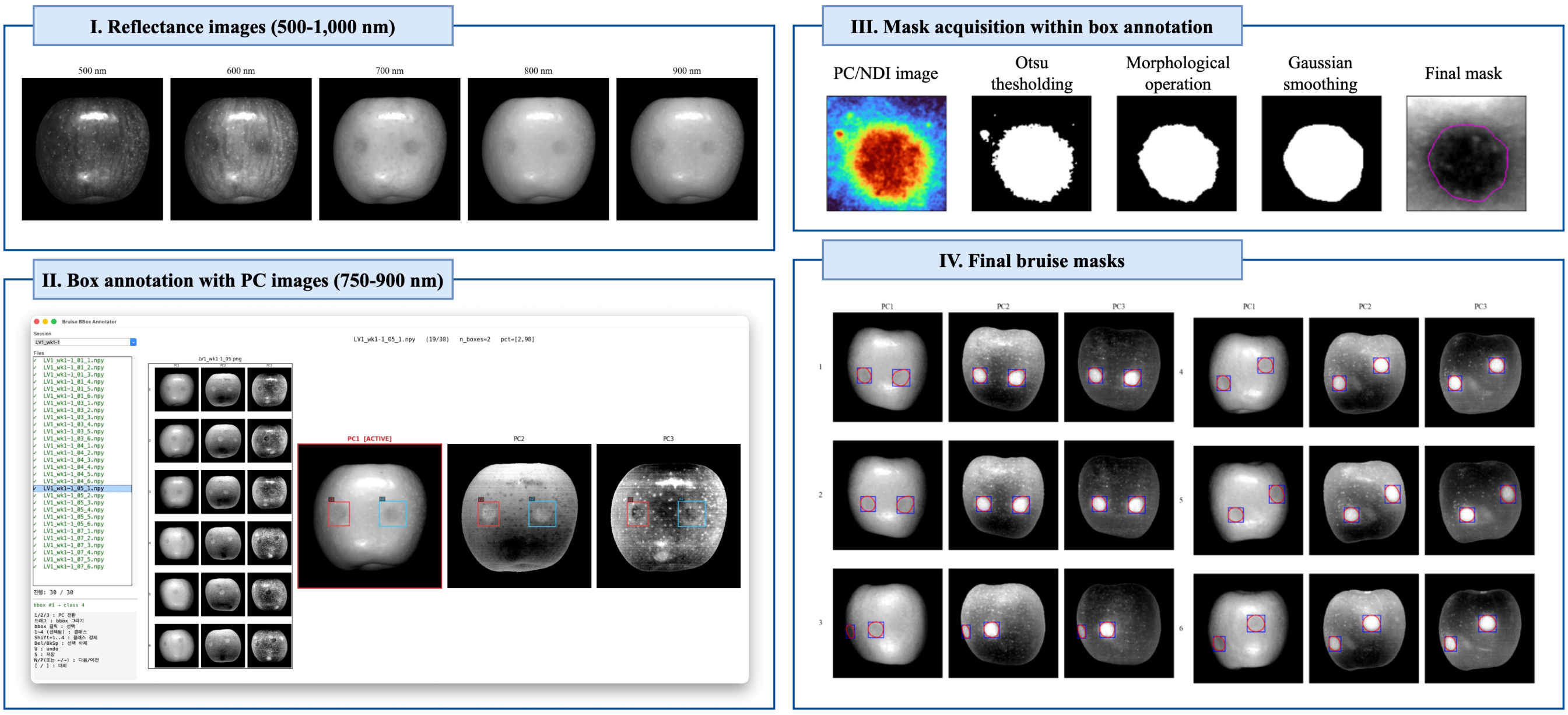

Supplementary Figure S1. Two-stage annotation workflow for bruise mask acquisition, performed with a manual box localization from PC score images followed by pixel-wise mask extraction from the selected PC/NDI contrast image.

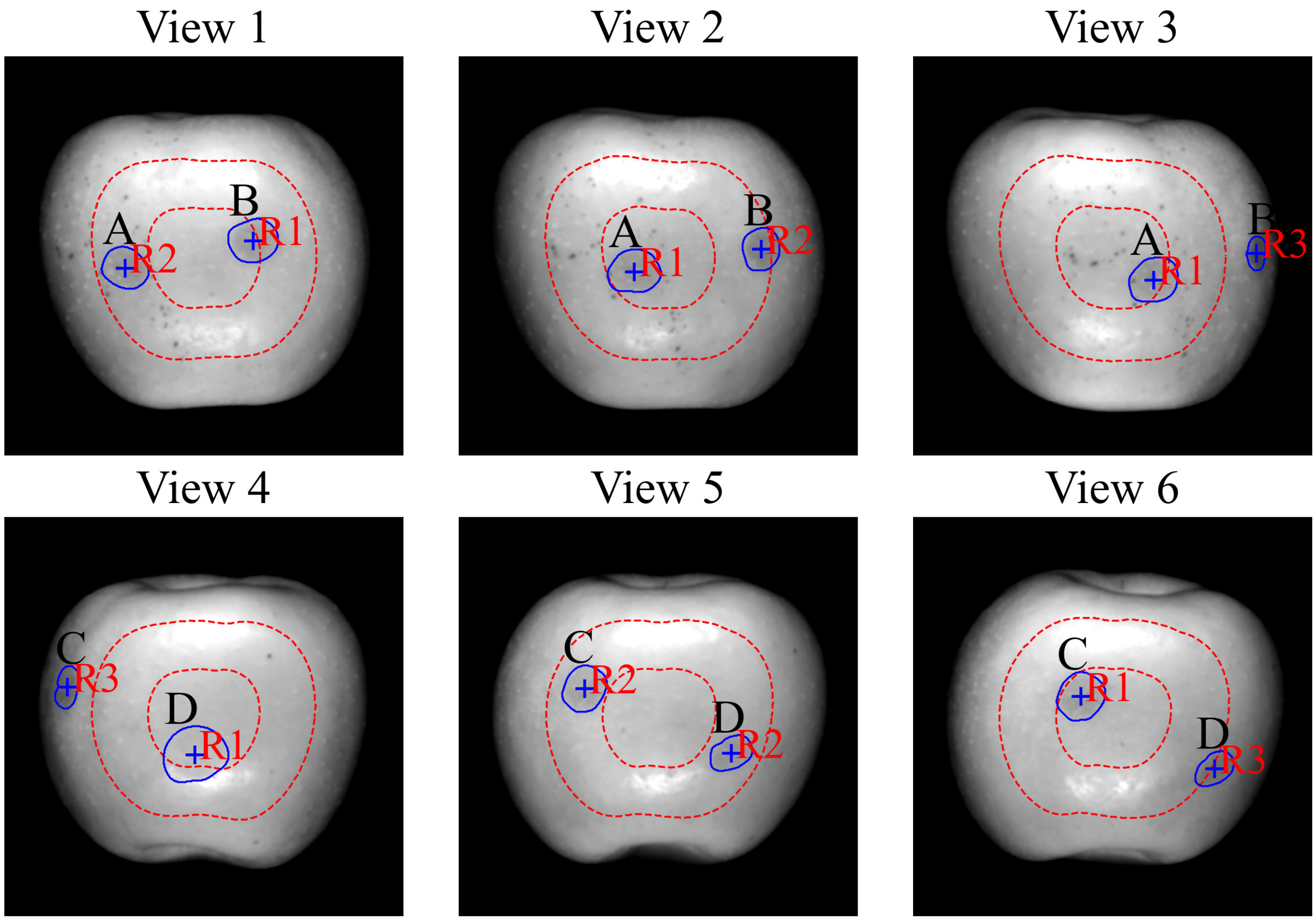

Supplementary Figure S2. Hyperspectral images of the same apple sample from six views acquired by rotating the fruit, illustrating the radial location categories assigned to each annotated bruise. Views 1–3 show the front side and views 4–6 the back side of the same apple. R1, R2, and R3 indicate the central, intermediate, and peripheral bruise-location categories, respectively.

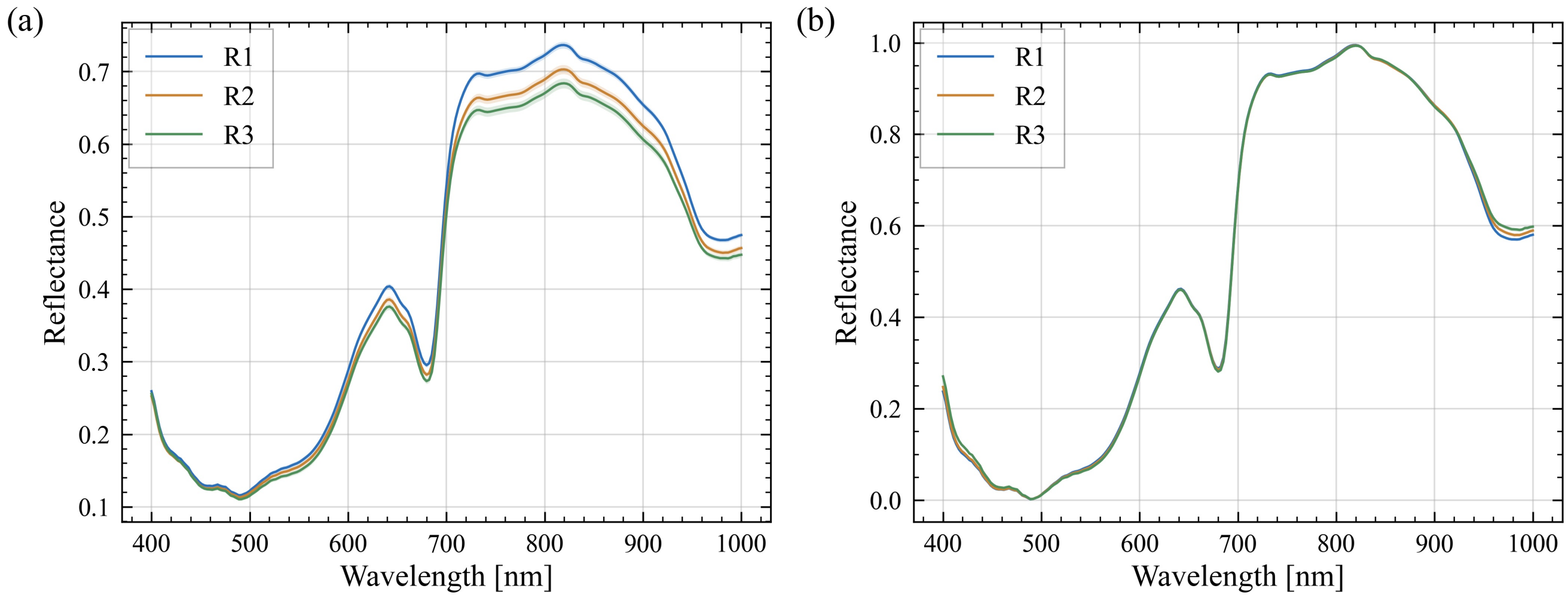

Supplementary Figure S3. Mean reflectance spectra of bruised regions grouped by radial location over all bruise instances. (A) Original spectra, and (B) spectra after sample-wise min-max normalization.

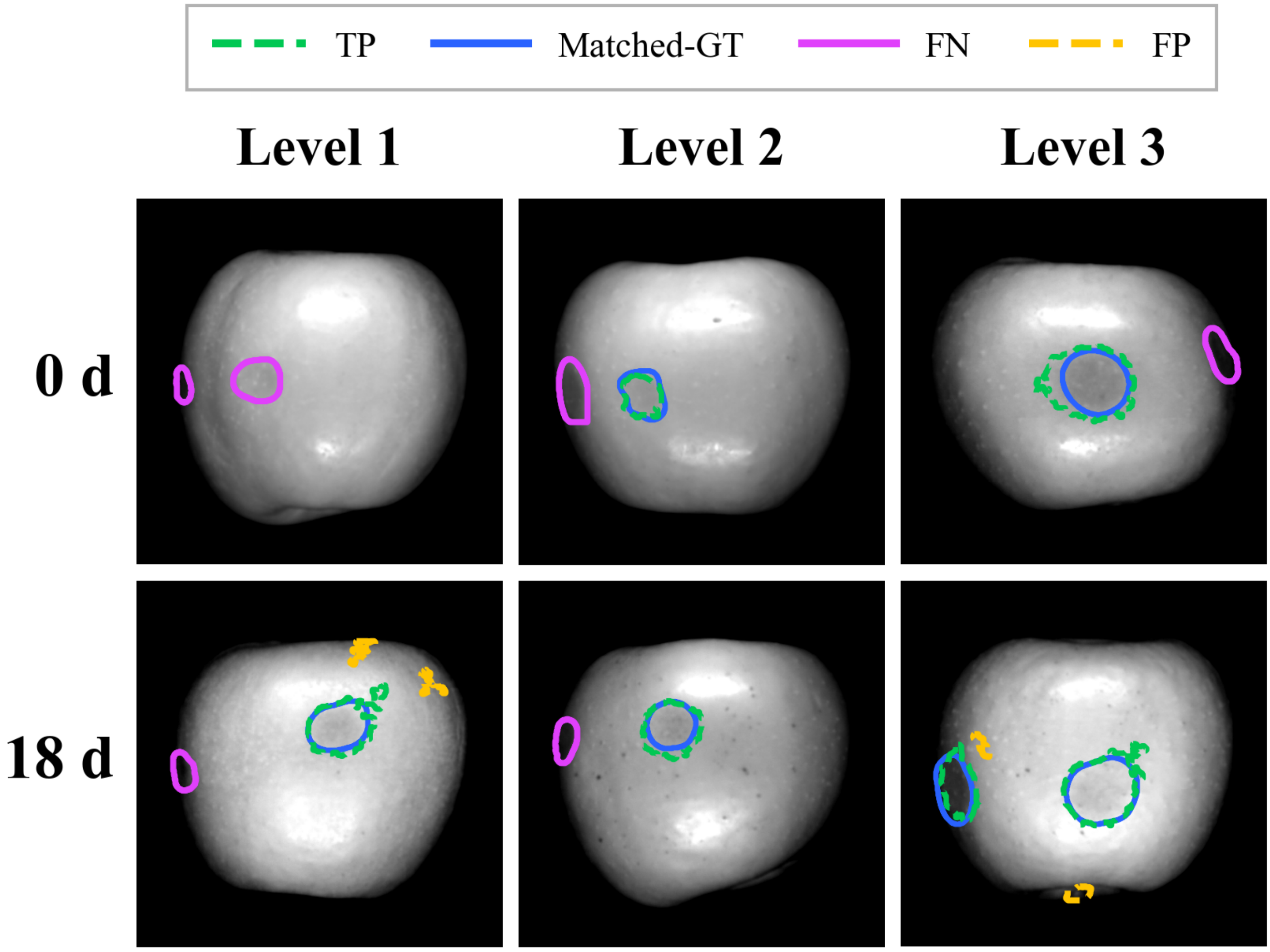

Supplementary Figure S4. Examples of bruise detection results obtained using the SVM model across impact level and storage day for the same samples shown in Figure 7.

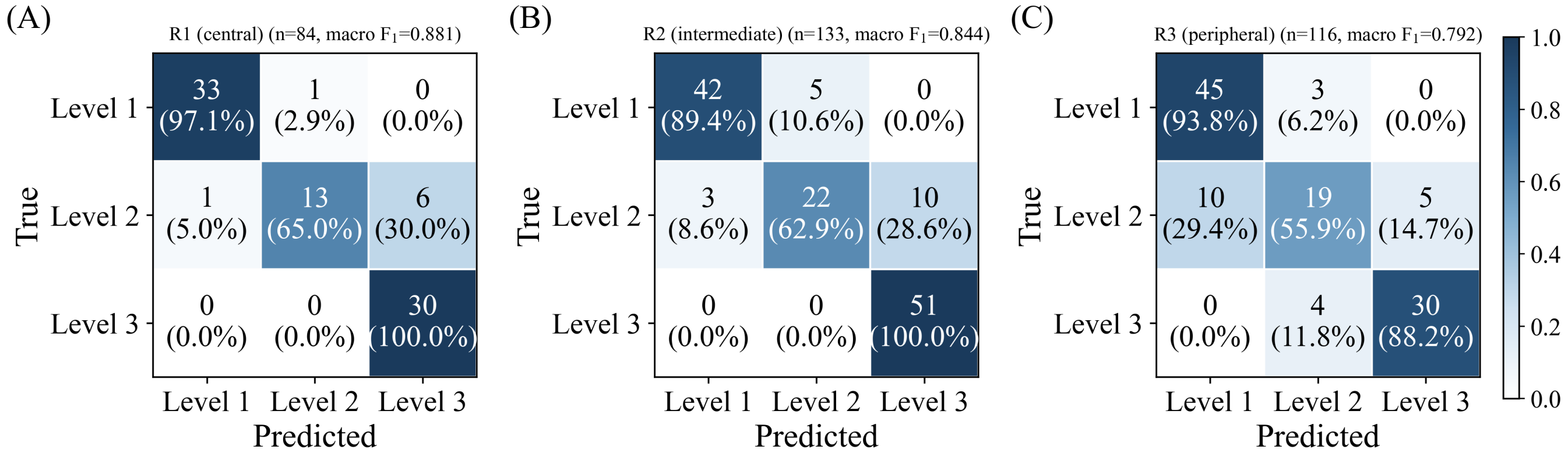

Supplementary Figure S5. Confusion matrices of impact level classification on the test subset for (A) R1, (B) R2, and (C) R3.

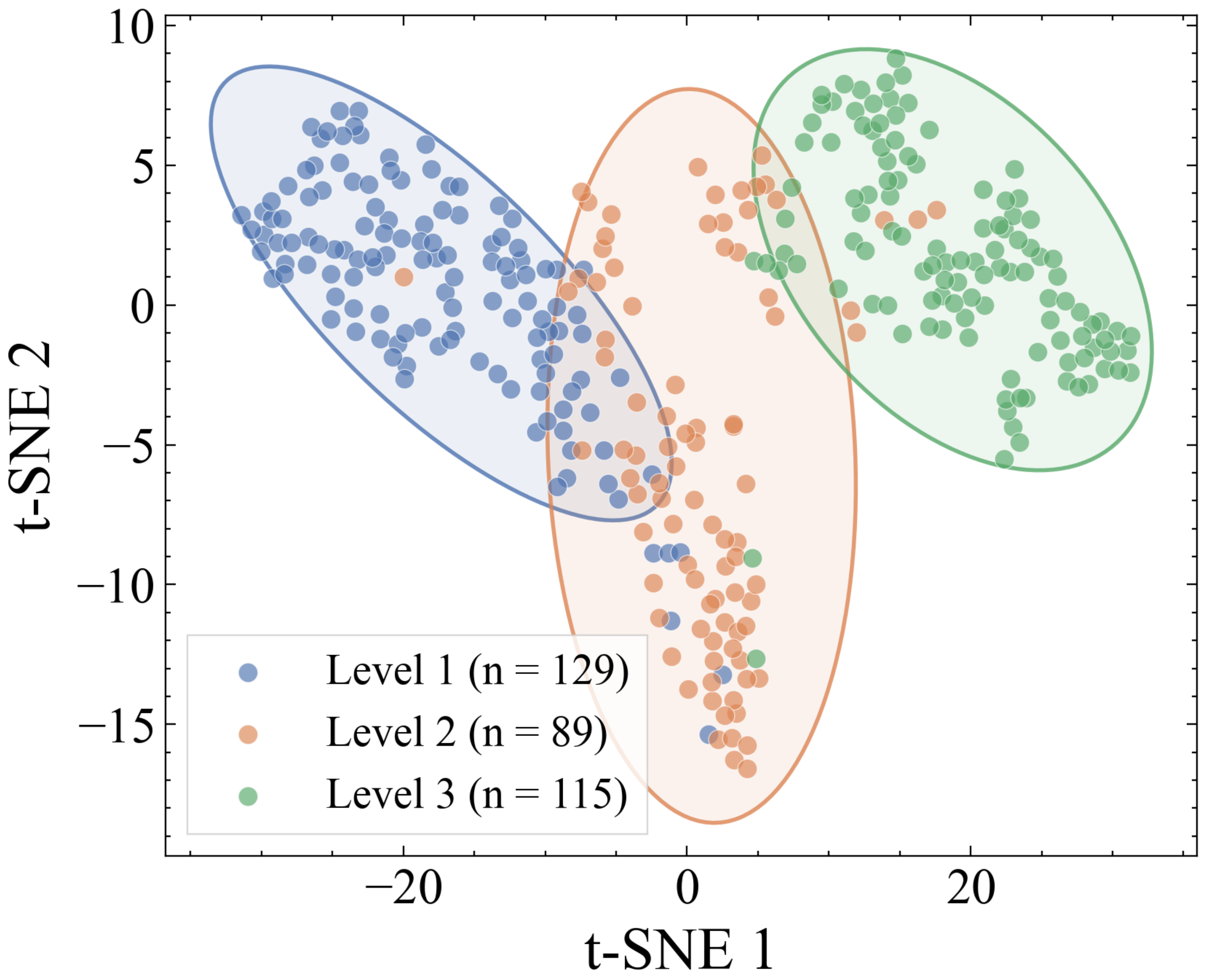

Supplementary Figure S6. t-SNE visualization of the final learned features of the impact level classifier for test bruise instances. Colors indicate impact levels, and shaded ellipses represent two standard deviations.

2 SUPPLEMENTARY TABLES

Supplementary Table S1. Summary of input datasets used for bruise detection, impact level classification, and cross-sectional bruise area regression models.

Modeling task	Input cube	Inclusion criterion	Total count	Training count	Validation count	Test count
Bruise detection	Entire image	Entire dataset	870 cubes / 1,676 masks	513 cubes / 987 masks	185 cubes / 356 masks	172 cubes / 333 masks
Impact-level classification	Bruise region	Entire dataset across all seven storage periods	1,676	987	356	333
Cross-sectional bruise-area regression	Bruise region	Bruise instances with cross-sectional RGB measurement of browning area	492	279	104	109

Supplementary Table S2. Number of bruise instances assigned to the central (R1), intermediate (R2), and peripheral (R3) location categories by impact level.

Impact level	R1	R2	R3	Total
Level 1	127	288	218	633
Level 2	95	229	174	498
Level 3	135	242	168	545
Overall	357	759	560	1,676

Supplementary Table S3. Bruise depth (mm) at each impact level and storage day. Values are mean ± SD, with n denoting the number of measurements. Within each storage day, mean values with different lowercase superscript letters differed significantly among impact levels ($p < 0.05$, one-way analysis of variance followed by Tukey's honestly significant difference test).

Impact level	1 d	4 d	8 d	11 d	15 d	18 d	Overall
Level 1	6.29 ± 1.06^{b} (n = 10)	6.44 ± 0.56^{b} (n = 10)	6.22 ± 1.16^{b} (n = 9)	5.92 ± 0.96^{b} (n = 9)	6.25 ± 0.66^{b} (n = 8)	5.65 ± 1.48^{b} (n = 10)	6.12 ± 1.03 (n = 56)
Level 2	7.03 ± 2.37^{ab} (n = 10)	6.96 ± 1.00^{b} (n = 9)	7.22 ± 1.10^{ab} (n = 7)	7.30 ± 1.74^{ab} (n = 10)	7.73 ± 0.82^{a} (n = 10)	7.77 ± 1.36^{a} (n = 10)	7.35 ± 1.48 (n = 56)
Level 3	8.49 ± 0.99^{a} (n = 10)	8.44 ± 1.40^{a} (n = 10)	8.60 ± 1.26^{a} (n = 8)	8.51 ± 2.59^{a} (n = 10)	7.97 ± 0.59^{a} (n = 8)	8.07 ± 1.22^{a} (n = 10)	8.35 ± 1.46 (n = 56)

Supplementary Table S4. Cross-sectional area (mm^2) of the bruises at each impact level and storage day. Values are mean ± SD, with n denoting the number of measurements. Statistical comparisons were performed after natural-log transformation. Within each storage day, mean values with different lowercase superscript letters differed significantly among impact levels ($p < 0.05$, one-way analysis of variance followed by Tukey's honestly significant difference test).

Impact level	1 d	4 d	8 d	11 d	15 d	18 d	Overall
Level 1	58.60 ± 13.62^{b} (n = 10)	58.84 ± 5.39^{b} (n = 10)	63.81 ± 13.32^{c} (n = 9)	56.54 ± 12.18^{c} (n = 9)	67.35 ± 13.23^{b} (n = 8)	53.60 ± 20.30^{b} (n = 10)	59.51 ± 13.86 (n = 56)
Level 2	71.83 ± 29.31^{b} (n = 10)	75.51 ± 16.43^{b} (n = 9)	86.00 ± 7.87^{b} (n = 7)	80.09 ± 11.95^{b} (n = 10)	83.27 ± 14.53^{b} (n = 10)	82.58 ± 15.59^{a} (n = 10)	79.63 ± 17.53 (n = 56)
Level 3	112.29 ± 21.59^{a} (n = 10)	110.67 ± 24.71^{a} (n = 10)	109.64 ± 17.58^{a} (n = 8)	112.90 ± 30.60^{a} (n = 10)	114.63 ± 18.34^{a} (n = 8)	104.74 ± 19.17^{a} (n = 10)	110.72 ± 21.95 (n = 56)

Supplementary Table S5. Bruise detection performance of the Hybrid 3D-2D U-Net with different input wavelength ranges.

Wavelength range (nm)	Spectral bands	Instance Dice	Box precision (@IoU 0.5)	Box recall (@IoU 0.5)	Box $F1$ (@IoU 0.5)
400–1,000	56	0.8431	0.9849	0.9760	0.9805
**600–900 (selected)**	**28**	**0.8607**	**0.9763**	**0.9850**	**0.9806**
400–600	18	0.7625	0.8559	0.8892	0.8722
900–1,000	9	0.7954	0.9485	0.9371	0.9428
